# Advanced Insights into Catalytic and Structural Features of the Zinc‐Dependent Alcohol Dehydrogenase from *Thauera aromatica*

**DOI:** 10.1002/cbic.202200149

**Published:** 2022-06-14

**Authors:** Frances Stark, Christoph Loderer, Mark Petchey, Gideon Grogan, Marion B. Ansorge‐Schumacher

**Affiliations:** ^1^ Professur für Molekulare Biotechnologie Technische Universität Dresden 01062 Dresden Germany; ^2^ Department of Chemistry University of York Heslington York YO10 5DD UK

**Keywords:** biocatalysis, enzyme catalysis, oxidoreductases, reduction, structure-activity relationships

## Abstract

The asymmetric reduction of ketones to chiral hydroxyl compounds by alcohol dehydrogenases (ADHs) is an established strategy for the provision of valuable precursors for fine chemicals and pharmaceutics. However, most ADHs favor linear aliphatic and aromatic carbonyl compounds, and suitable biocatalysts with preference for cyclic ketones and diketones are still scarce. Among the few candidates, the alcohol dehydrogenase from *Thauera aromatica* (ThaADH) stands out with a high activity for the reduction of the cyclic α‐diketone 1,2‐cyclohexanedione to the corresponding α‐hydroxy ketone. This study elucidates catalytic and structural features of the enzyme. ThaADH showed a remarkable thermal and pH stability as well as stability in the presence of polar solvents. A thorough description of the substrate scope combined with the resolution and description of the crystal structure, demonstrated a strong preference of ThaADH for cyclic α‐substituted cyclohexanones, and indicated structural determinants responsible for the unique substrate acceptance.

## Graphical abstract

**The zinc‐dependent MDR−ADH** (medium chain dehydrogenase/reductase−alcohol dehydrogenase) from *Thauera aromatic*a has a unique substrate preference for sterically demanding α‐substituted linear cyclic carbonyl compounds and a remarkable thermal, pH and solvent stability. Based on high‐resolution structures from X‐ray analysis, structural determinants for the enzyme's special features are introduced.

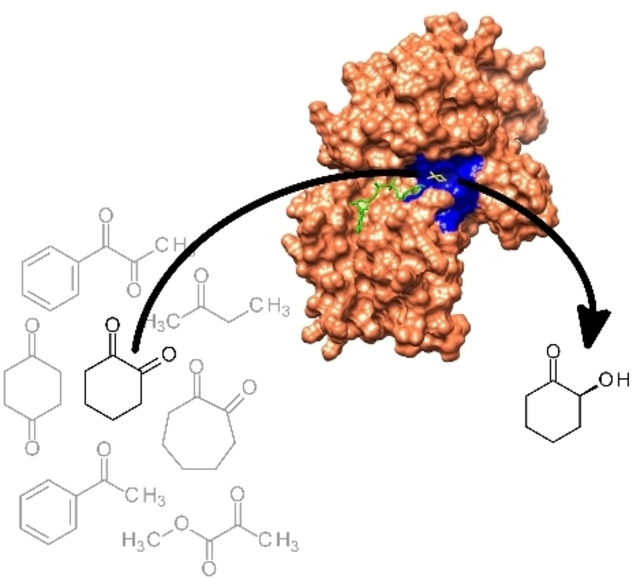

## Introduction

Of the top 200 small molecule drugs listed by the University of Arizona in 2020,^[1]^ more than 60 % are chiral compounds. In many cases, their production strongly relies on the introduction of chiral secondary alcohols as a main building block.^[2]^ A widespread strategy for the synthesis of these molecules is the asymmetric reduction of prochiral ketones using alcohol dehydrogenases (ADHs) as selective catalysts.[^3a^, ^3b^, ^3c^] Among these are many zinc‐dependent ADHs of the medium chain dehydrogenase/reductase (MDR) superfamily such as the well‐known ADH from horse liver (HLADH) and the carbonyl reductase from *Candida parapsilosis* (CPCR2).

MDR−ADHs share a high structural similarity.[^4a^, ^4b^] Nevertheless, they reveal vastly different biochemical features, *e. g*. regarding thermal stability[^5a^, ^5b^] or chemo‐tolerance.^[6]^ In stereochemical terms, they follow Prelog's rule almost without exception;^[7]^ their chemo‐, regio‐, and stereoselectivity is often remarkable. As a whole, MDR−ADHs are able to convert a large spectrum of carbonyl substrates ranging from short chain aldehydes and ketones to bulky cyclic compounds.[^8a^, ^8b^, ^8c^] A widely accepted model of the substrate‐binding site explains this with the accommodation of the two side chains of the reactive carbonyl group in a large and a small hydrophobic binding pocket, respectively, determining substrate orientation.^[9]^ In accordance, most MDR−ADHs clearly prefer linear aliphatic and phenyl substituted carbonyls as substrates. A few MDR−ADHs also reduce cyclic non‐aromatic ketones, mostly cyclohexanone and its methylated derivatives, albeit at a comparatively slow rate.[^5a^, ^8a^, ^8b^, ^8c^, ^10a^, ^10b^] In the case of HLADH, the range of cyclic ketones acting as substrates is rather broad.[^11a^, ^11b^, ^11c^] Nevertheless, substrate acceptance rarely expands to sterically highly demanding cyclic compounds such as the α‐diketone 1,2‐cyclohexanedione.[^12a^, ^12b^] However, the asymmetric reduction of prochiral α‐diketones is of particular interest, because it can result in the formation of chiral α‐hydroxy ketones, which again are important building blocks for chemical synthesis.[^13a^, ^13b^, ^13c^, ^13d^]

A few oxidoreductases have been described to catalyze the reduction of cyclic α‐diketones. The NADPH‐dependent ADH−T from *Thermoanaerobacter* sp. converted 1,2‐cyclohexanedione, but with a 20‐fold lower activity than small arylaliphatic ketones and aldehydes such as acetone and acetaldehyde, respectively.^[14]^ Cyclohexanol dehydrogenase from *Pseudomonas* sp. K 601 also showed low conversion of 1,2‐cyclohexanedione, but higher activities with structurally similar substrate molecules such as 1,4‐cyclohexanedione.^[15]^ A ketoreductase domain of the mycolactone biosynthesis reduced 1,2‐cyclohexanedione with low catalytic efficiency.^[16]^ Whole cells of *Candida parapsilosis* ATCC 7330 were reported to reduce 1,2‐ as well as 1,4‐cyclohexanedione, but the responsible biocatalyst was not identified.^[17]^ *Serratia marcescens* CECT 977 2,3‐butanediol dehydrogenase reduced 1,2‐cyclohexanedione as well as 1‐phenyl‐1,2‐propanedione, but with significantly lower activity than linear aliphatic α‐diketones.^[12b]^ On the other hand, two NADPH‐dependent diacetyl reductases from baker's yeast with considerable activities towards the reduction of 1,2‐cyclohexanedione were reported.^[18]^ To the best of our knowledge, the only native NADH‐dependent MDR−ADH with a clear preference for conversion of cyclic α‐diketones still is the alcohol dehydrogenase from *Thauera aromatica* (ThaADH), which we introduced in a previous publication.^[19]^ It selectively reduces 1,2‐cyclohexanedione to the corresponding α‐hydroxy ketone.

In this study, we present further insight into biochemical features of ThaADH, which bear relevance for synthetic use, and look to elucidate structural reasons for the rather unique substrate scope of this enzyme. In this context, we determined the X‐ray crystallographic structure of the wild type (WT) ThaADH in complex with NADH, refined to a resolution of 2.60 Å, as well as of the double mutant K319A/K320A without cofactor, refined to 1.80 Å.

## Results and Discussion

### Effects of temperature, pH and polar solvents on enzyme activity and stability

**Figure 1:**
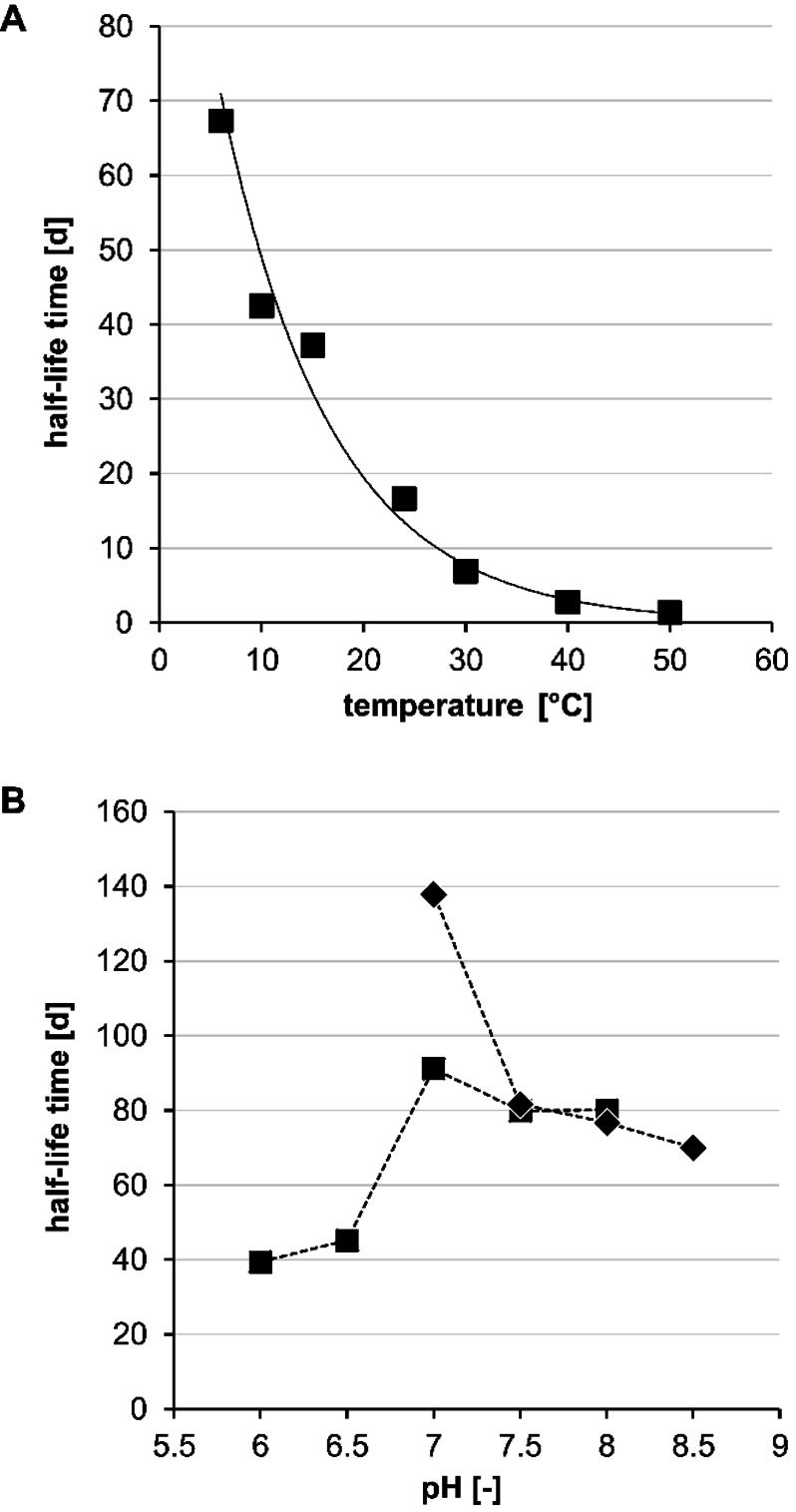
Stability of ThaADH with respect to temperature (A) and pH (B). Stability was determined by measuring the residual enzyme activity after incubation at the respective temperatures and pH values for various time periods. All measurements were performed in triplicate. Activities refer to the reduction of 1,2‐cyclohexanedione. A: Enzyme was incubated in 0.1 mmol L^−1^ TEA, 150 mmol L^−1^ NaCl, pH 7.5. B: Enzyme was incubated at room temperature, squares: potassium phosphate buffer; rhombs: TEA buffer. TEA: triethanolamine.

The dependence of the activity of ThaADH on temperature was described in detail in our previous publication.^[19]^ We determined the highest reaction rate at 50 °C, and observed rapid precipitation of the enzyme above this temperature. Here, further investigation of the thermal stability of ThaADH revealed an exponential dependence of the half‐life times (t_1/2_) on the temperature (Figure 1A). At the lowest investigated temperature of 6 °C, t_1/2_ was 67 d; at room temperature (25 °C) t_1/2_ was 4‐fold lower (17 d), and at the maximum temperature (50 °C) t_1/2_ was 31 h.

In accordance with our previous findings, we found a sharp pH‐optimum for the reductive activity of ThaADH,^[19]^ but were able to refine the absolute value to pH 6.5. The highest oxidative activity was obtained at a more alkaline pH of 9.0 (Figure S1). The enzyme was sufficiently stable over the entire pH range between 6.0 and 8.5 (Figure 1B). Stability was best at pH 7.0, but strongly depended on the buffer salt employed. With triethanolamine (TEA), t_1/2_ was 138 d, while it was only 91 d with potassium phosphate. The stability decrease was slower in alkaline than in acidic media.

**Figure 2:**
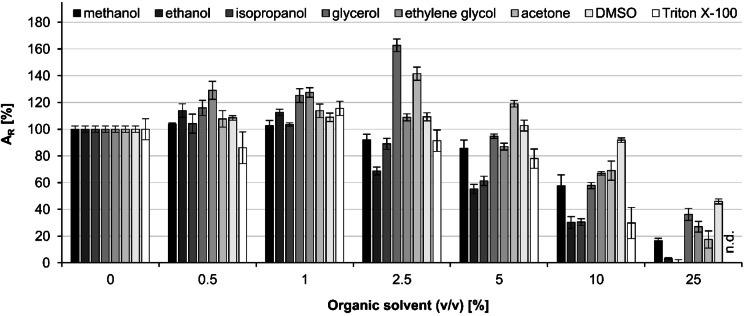
Activity of ThaADH in the presence of water‐miscible organic solvents at different volume ratios. Activities refer to the reduction of 1,2‐cyclohexanedione. Activity without solvent was set as 100 % and is equivalent to 14.5 U mg^−1^. Error bars indicate the standard deviation of three independent measurements. A_R_: relative activity, n.d.: not detectable.

**Figure 3:**
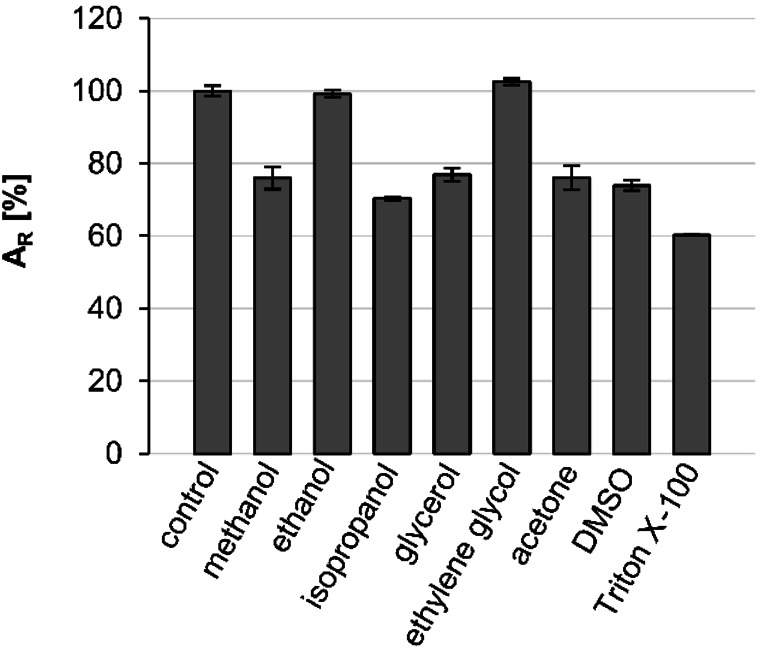
Stability of ThaADH in 20 % (v/v) polar organic solvents. Stability was determined by measuring the residual activity after incubation with organic solvents at room temperature for 4 d. Activities refer to the reduction of 1,2‐cyclohexanedione. Activity of incubation without solvent was set as 100 % and is equivalent to 21.8 U mg^−1^. Error bars indicate the standard deviation of three independent measurements. A_R_: relative activity.

Finally, we investigated the influence of frequently employed water‐miscible organic solvents on ThaADH activity (Figure 2) and stability (Figures 3), since the synthetic use of enzyme catalysts often requires addition of such solvents to provide appropriate substrate concentrations. For most of the investigated solvents, an increasing concentration resulted in an initial activity increase (up to 5 % volume fraction) followed by a clear decrease (more than 10 % volume fraction). The increase was apparent with Triton X‐100 (1 % (v/v)), ethanol (0.5 % and 1 % (v/v)), glycerol, ethylene glycol and dimethyl sulfoxide (DMSO) (0.5 %, 1 % and 2.5 % (v/v)) and acetone (0.5 %, 1 %, 2.5 % and 5 % (v/v)). The strongest subsequent decrease occurred with ethanol, isopropanol, and Triton‐X‐100, where the relative activity dropped to about 30 % at a volume ratio of 10 % of each solvent. Activity was least impaired by DMSO, with which we obtained a relative enzyme activity of 92 % at a solvent ratio of 10 % (v/v), and even an activity of 46 % at a ratio of 25 % (v/v).

In various solvents, ThaADH remained active over 4 d. With 20 % (v/v) ethanol or ethylene glycol, no activity decrease occurred at all. In the presence of methanol, isopropanol, glycerol, acetone and DMSO, the activity loss was between 23 % and 30 %. Only treatment with Triton X‐100 diminished the activity by 40 %.

Compared to other published ADHs, the observed activities and stabilities of ThaADH under synthetically relevant conditions are rather promising. As expected, activity^[19]^ and stability towards temperature behave inversely. However, ThaADH shows t_1/2_ in the range of 67 d (at 6 °C) to 31 h (at 50 °C) (Figure 1A), which is considerably higher than those of other ADHs from mesophile organisms.[^6^, ^20a^, ^20b^, ^20c^] The ADHs from *Leifsonia* sp. strain S749 and *Brevibacterium* sp. KU 1309, for example, show a residual activity of about 80 % after an incubation at 40 °C for 0.5 or 1 h, respectively,[^21a^, ^21b^] while ThaADH shows a residual activity of 88 % after incubation at the same temperature for 1 d.

With regard to pH‐stability, ThaADH performs similarly well. Here, the enzyme shows t_1/2_ between 39 d (at pH 6.0) and 138 d (at pH 7.0) (Figure 1B), while those of other ADHs are in the range of only hours,[^20b^, ^20c^] and overall in a more alkaline milieu.[^20a^, ^20c^, ^22a^, ^22b^]

The observed activity increase of ThaADH in the presence of low concentrations of water‐miscible organic solvents (Figure 2) is in accordance with other reports in the literature.[^23a^, ^23b^, ^23c^] The explanation offered was the influence of the solvent on the hydrate shell of the enzyme, resulting in improved hydration. The disruption of this hydrate shell was held responsible for the activity decrease at higher volume fractions of the solvents observed with ThaADH.^[24]^ The effect has also been reported for a wide variety of oxidoreductases, yet with marked differences for the individual solvents. For instance, the ADH from *Thermococcus kodakarensis* (TkADH) showed at least twofold higher residual activities with 20 % (v/v) of methanol, ethanol, and isopropanol, respectively, than ThaADH with 25 % (v/v) of the same solvents. In contrast, with acetone and DMSO, the residual activities of ThaADH were similar or higher, respectively.^[25]^ Compared to the thermostable ADH from *Thermus* sp. ATN1, ThaADH shows similar residual activities with ethanol at 10 % (v/v), but with methanol, isopropanol, acetone and DMSO (10 % v/v each), the residual activities of ThaADH are up to 4.5‐fold higher.^[8a]^

Likewise, ThaADH‐stability depended on the type of solvent (Figure 3). A comparatively high residual activity ranging from 60 % (with Triton X‐100) to 100 % (with ethylene glycol) was observed after 4 d incubation. TkADH showed slightly higher residual activities in methanol, ethanol, isopropanol, acetone, and DMSO, but only after a relatively short incubation over 4 h in 20 % (v/v) solvent.^[25]^ The glyceraldehyde dehydrogenase from *Thermoplasma acidophilum* showed a residual activity of about 40 % after only 30 min incubation in also 20 % (v/v) ethanol,^[26]^ while ThaADH hardly lost any activity within the same time.

### Substrate specificity and reaction characteristics

**Figure 4:**
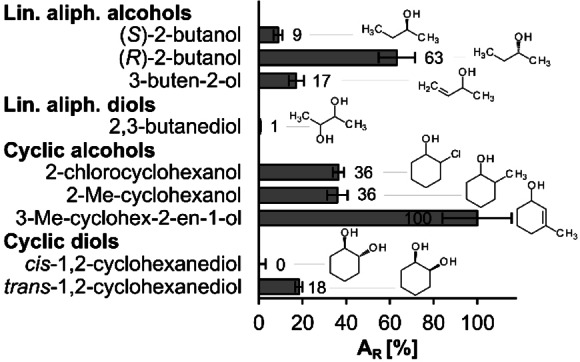
Substrate specificity of ThaADH for oxidation reactions. Activity with 3‐methylcyclohex‐2‐en‐1‐ol was set as 100 % and is equivalent to 0.9 U mg^−1^. Error bars indicate the standard deviation of three independent measurements. A_R_: relative activity, Me: methyl.

**Figure 5:**
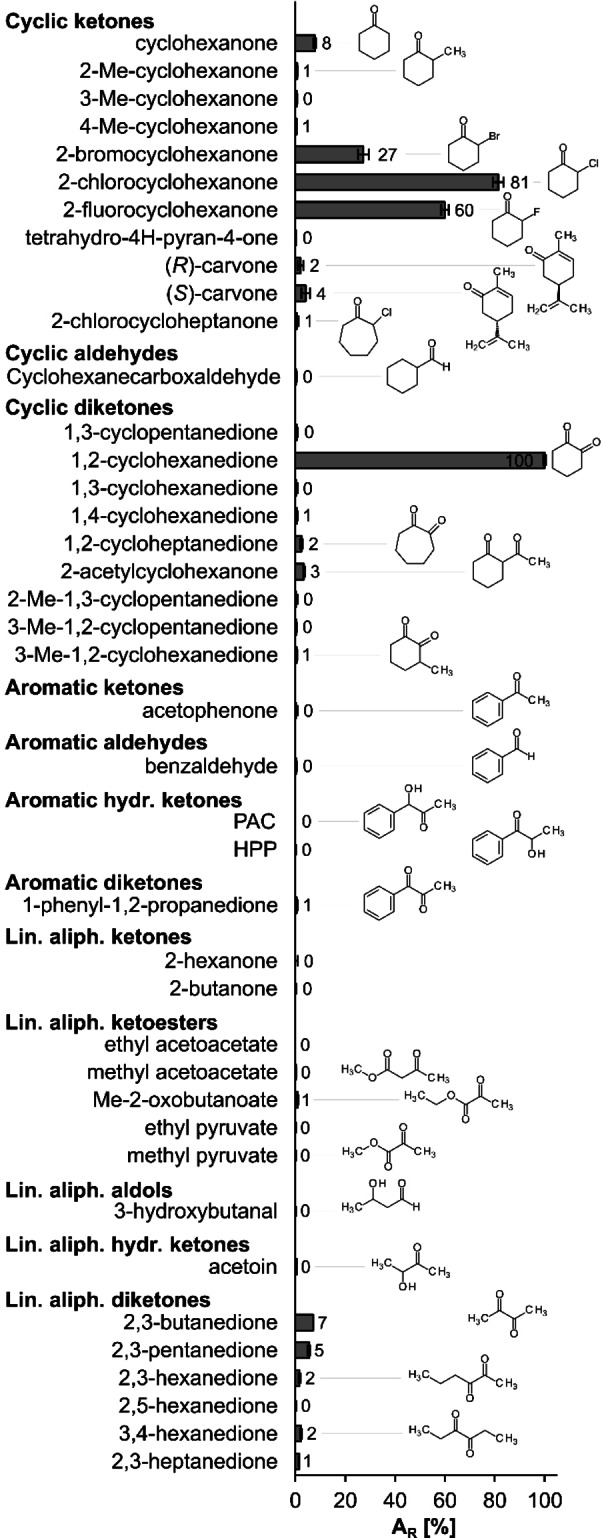
Substrate specificity of ThaADH for reduction reactions. Activity with 1,2‐cyclohexanedione was set as 100 % and is equivalent to 6,3 U mg^−1^. Error bars indicate the standard deviation of three independent measurements. A_R_: relative activity, Me: methyl, PAC: phenylacetylcarbinol, HPP: 2‐hydroxy‐1‐phenyl‐1‐propanone.

Based on our previous observations on the reaction scope of ThaADH,^[19]^ we conducted a detailed examination of the oxidative (Figure 4) as well as reductive (Figure 5) activities. Regarding substrate oxidation, 3‐methylcyclohex‐2‐en‐1‐ol was converted most rapidly with a specific activity of 0.9 U mg^−1^. Considering only cyclic compounds, 2‐chloro‐ and 2‐methylcyclohexanol as well as *trans*‐ and *cis*‐1,2‐cyclohexanediol followed with decreasing reaction rates. However, ThaADH also converted some linear aliphatic molecules with good activity. (*R*)‐2‐butanol was oxidized seven times faster than (*S*)‐2‐butanol. The corresponding alkenol 3‐butene‐2‐ol was converted as well.

Regarding substrate reduction, ThaADH was most active on 1,2‐cyclohexanedione, which is in accordance with our previous findings.^[19]^ The specific activity for this substrate was 6.3 U mg^−1^ and thus 7‐fold higher than the fastest observed reaction rate for oxidation. Among the cyclic carbonyl compounds, α‐halogenated cyclohexanones and cyclohexanone were converted with decreasing reaction rates. In contrast, methylated cyclohexanones (2‐, 3‐ and 4‐methylcyclohexanone) as well as diketones whose carbonyl groups were not in the α‐position (1,3‐ and 1,4‐cyclohexanedione) were not accepted. Likewise, ThaADH hardly converted cyclic ketones and diketones with a ring structure of five or seven carbon atoms. 2‐Acetylcyclohexanone and carvone were converted moderately, whereas (*S*)‐carvone was reduced two times as fast as the (*R*)‐enantiomer. The one cyclic aldehyde that was tested, cyclohexanecarboxyaldehyde, underwent no reduction. ThaADH also did not reduce any compounds with aromatic substituents, such as acetophenone and benzaldehyde. Among the linear aliphatic carbonyl compounds, only diketones underwent a small conversion. 2,3‐butanedione and 2,3‐pentanedione were reduced most rapidly, 2,3‐hexanedione and 3,4‐hexanedione considerably more slowly.

Kinetic parameters were determined for substrates with remarkable reaction rates (Table 1), usually at 40 °C. With 1,2‐cyclohexanedione and 2‐bromo‐ and 2‐chlorocyclohexanone data were obtained at room temperature for experimental reasons. For all substrates, the Michaelis‐Menten constants (*K*_M_) were above 20 mmol L^−1^. The catalytic efficiency (*k*_cat_/*K*_M_) was in the range of 0.08 to 0.57 s^−1^ mmol L^−1^. For the co‐substrate a 14‐fold lower *K*_M_ was detected with NADH than with NAD^+^. The catalytic efficiency with NADH was even 58 times higher than with the oxidized form. The synthetic NAD^+^ analogue carba‐NAD^+^ (cNAD^+^) was accepted with comparable affinity to NAD^+^.

**Table 1:** Kinetic parameters of substrate conversion with ThaADH. Error bars indicate the standard deviation of three independent measurements.

Substrate	v_max_ [U mg^−1^]	*K*_M_ [mmol L^−1^]	*k*_cat_ [s^−1^]	*k*_cat_/*K*_M_ [s^−1^ mmol L^−1^]
3‐methylcyclo‐hex‐2‐en‐1‐ol	2.4 ±0.1	21.6 ±1.9	1.77 ±0.05	0.08 ±0.01
1,2‐cyclohexanedione^[a]^	53.3 ±13.8	68.1 ±33.8	39.11 ±10.13	0.57 ±0.43
2‐bromocyclohexanone^[a,b]^	6.3 ±0.6	24.1 ±6.8	4.62 ±0.47	0.19 ±0.07
2‐chlorocyclohexanone^[a,c]^	14.9 ±1.9	33.1 ±10.4	10.93 ±1.38	0.33 ±0.15
NAD^+^	2.2 ±0.1	1.0 ±0.2	1.63 ±0.10	1.63 ±0.43
cNAD^+^	0.9 ±0.1	0.7 ±0.1	0.66 ±0.04	0.94 ±0.19
NADH	9.1 ±1.0	0.07 ±0.02	6.63 ±0.74	94.74 ±37.63

[a] Room temperature. [b] 3 % (v/v) DMSO. [c] 1 % (v/v) DMSO. Nonlinear regressions of the kinetic data are provided in (Figure S2).

**Scheme 1:**
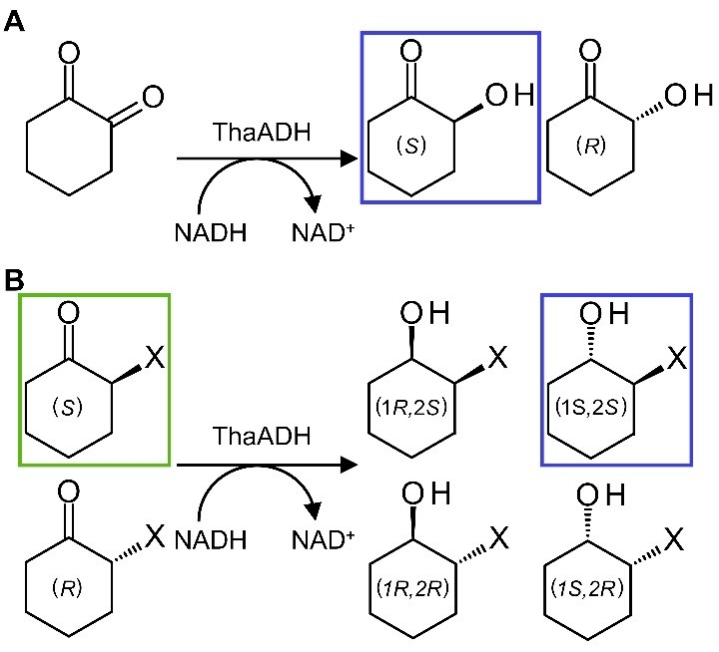
Stereospecificity and stereoselectivity of ThaADH in the reduction of 1,2‐cyclohexanedione (A) and halogenated cyclohexanone (B). The preferred substrate enantiomer is highlighted in green, preferred product enantiomers are highlighted in blue.

In accordance with our previous findings, ThaADH selectively mono‐reduced 1,2‐cyclohexanedione to the corresponding α‐hydroxy ketone 2‐hydroxycyclohexanone (Scheme 1A); diol formation was not observed.^[19]^ The preferred reaction product was (*S*)‐2‐hydroxycyclohexanone with an ee of 75 % (Table 2). With the chiral substrates 2‐bromo‐ and 2‐chlorocyclohexanone, the (*S*)‐configured compounds were clearly preferred (Scheme 1B). Analysis of the products with ^1^H‐NMR (Figures S6–11), polarimetry (Table S1) and GC‐MS (Figures S12–19) showed the *trans*‐configured alcohols, (1*S*,2*S*)‐bromo‐ and chlorocyclohexanol with a de of 88 % and 81 %, respectively, as the major outcome of the conversions (Scheme 1B, Table 2). Thus, apparently ThaADH was (*S*)‐selective in all three cases. Unfortunately, we were not able to investigate the behavior towards the fluorinated substrate as the (probably) resulting enantiomers underwent immediate racemization.

**Table 2:** Stereospecificity and Stereoselectivity with enantiomeric/diastereomeric excess of ThaADH.

Substrate	Substrate preference	Product preference	ee/de [%]
1,2‐cyclohexanedione	–	(*S*)–	75
2‐bromocyclohexanone	(*S*)–	(S,S)–	88 trans–
2‐chlorocyclohexanone	(*S*)–	(S,S)–	81 trans–

The results obtained confirm the previously observed preference of ThaADH for cyclic carbonyl compounds over aromatic and linear aliphatic compounds (Figure 4 and 5). This preference is in line with the physiological function of the enzyme, *i. e*. the oxidation of the cyclic 6‐hydroxycyclohex‐1‐ene‐1‐carboxyl‐CoA within the benzoyl‐CoA metabolism.^[27]^ However, both oxidation rate (Figure 4) and corresponding *K*_M_ (Table 1) in our study are far below that of the physiological oxidation (A_S_=11.8 U mg^−1^, *K*_M_=60±20 μmol L^−1^).^[27]^ The low affinity of the biocatalyst to all substrates tested suggests a crucial role of the CoA‐substituent for substrate binding. However, since the solubility limits of the substrates were reached in our study, despite addition of DMSO, the kinetic parameters should be interpreted with caution. In contrast to the physiological role of ThaADH, we found a 14‐fold lower *K*_M_ and approximately 60‐fold higher catalytic efficiency with NADH than with NAD^+^ (Table 1).

In line with the structure of the physiological substrate, a conjugated double bond at the Cα of the reactive group‐bearing carbon atom as with 3‐methylcyclohex‐2‐en‐1‐ol and the carbonyl group within a cyclohexyl‐structure seems to be beneficial for a fast conversion by ThaADH. The relative position of substituents to the reactive group also seems to be important, which is shown by the reduction of 1,2‐cyclohexanedione, but not 1,3‐ and 1,4‐cyclohexanedione. According to the four most rapidly reduced carbonyl compounds in this work, an electron‐withdrawing effect (positive inductive/mesomeric effect), mediated by a second carbonyl function or halogen atom in α‐position, improves substrate binding. In the reduction process, the reactive carbonyl oxygen lends a pair of electrons to the catalytic zinc, which decreases the electron density of the carbonyl group.^[28]^ Additional reduction of the electron density in the reactive carbonyl function, caused by electron‐withdrawing substituents seems to favor the uptake of the hydride ion. This agrees with observations of a favorable impact of substituents with negative inductive effect on the reduction of neighboring carbonyl functions described in other studies.[^29a^, ^29b^] Conversely, substituents with a negative inductive effect are disadvantageous for oxidation,^[30]^ and electron‐shifting substituents, such as a methyl group, seem to be disadvantageous for reduction, albeit indiscriminating for oxidation. However, reduction of simple cyclohexanone by ThaADH shows that a substituent in the preferred α‐position is not essential for the reduction of a substrate molecule. Nevertheless, α‐diketones are the preferred substrates of ThaADH.

If the carbonyl group is located within a cyclopentyl or cycloheptyl structure, or if an aromatic substituent is present at the carbonyl function, reduction with ThaADH is not possible. This contrasts with the substrate acceptance of many other well‐known ADHs, such as CPCR2.[^8c^, ^31^] The conversion of linear aliphatic diketones with much lower rates does not contradict these observations, since such molecules could adopt a ring‐like structure and thus achieve binding similar to cyclic compounds. However, the reduction rate decreased with an increasing chain length.

**Scheme 2:**
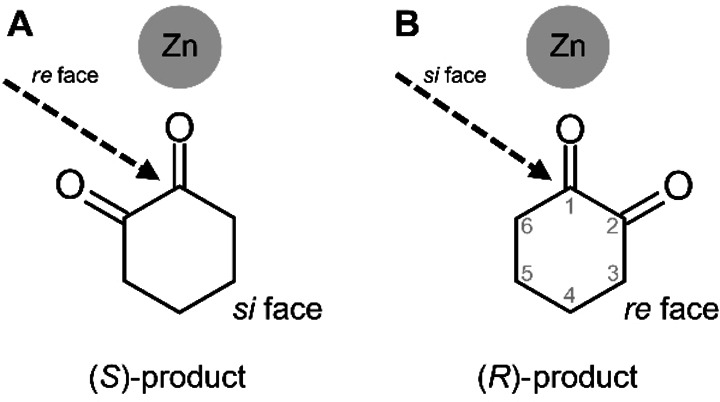
Schematic position of substrate 1,2‐cyclohexanedione in the active site of ThaADH depending on the carbonyl group bound to the catalytic zinc.

Substrate acceptance of ThaADH is clearly stereospecific as observed for the oxidation of trans‐1,2‐cyclohexanediol and (*R*)‐2‐butanol (Figure 4), as well as for the reduction of 2‐bromo‐ and 2‐chlorocyclohexanone (Table 2, Scheme 1). However, ee and de reveal that the (*S*)‐selectivity is not exclusive. In the reduction of 1,2‐cyclohexanedione the transfer of the pro‐*R* hydrogen of NADH to the *re*‐face is preferred, yielding the (*S*)‐product according to Prelog's rule. This agrees with the activity of most MDR−ADHs. However, depending on which of the two carbonyl groups orient towards the zinc, hydride transfer from the *si* face and thus formation of the (*R*)‐product is also possible (Scheme 2). Hence, the enantioselectivity is as much an expression of the regiospecificity of ThaADH in the reduction of 1,2‐cyclohexanedione. The responsible structural features of ThaADH for substrate acceptance, we elucidate in the following section.

### Structural features

In our previous study, we determined the molecular weight of the recombinant, Strep‐tagged monomer subunit of ThaADH as about 40 kDa.^[19]^ Here, size exclusion chromatography (SEC) showed the enzyme in solution exclusively as a dimer with a molecular weight of 89 kDa (Figure S3), which is in good agreement with the description of the dimeric structure of the native enzyme with a molecular mass of 78000±10000 kDa.^[27]^ On the other hand, blue native PAGE documented tetrameric and higher oligomeric states (Figure S5). In agreement with this observation, the proposed dependency of the predominant oligomeric state of zinc‐dependent MDRs on the length of the quaternary structure‐determining loop (QSDL)^[32]^ postulates ThaADH as a tetramer. This prediction is consistent with the fact that prokaryotic ADHs occur as tetramers in most cases.

**Figure 6:**
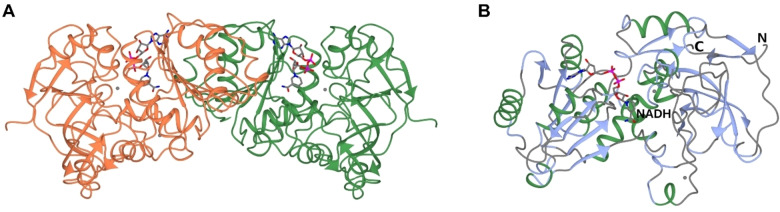
Structure of dimer (A) and monomer (B) of ThaADH in ribbon format. NADH is shown in cylinder format with carbon atoms in grey; zinc atoms are shown as grey spheres. A: monomers shown in coral and green.

In order to shed further light on structural features, the crystal structure of ThaADH was solved in two forms: First, the WT enzyme in complex with NADH, which had 3 molecules in the asymmetric unit (asu), comprising one‐and‐a‐half dimers, and was refined to a resolution of 2.60 Å. The quality of the model was good in monomers A and B but electron density was poorer for monomer C leading to some unmodelled side chains and higher *B* factors for this molecule. Second, the K319A/K320A double mutant, which was obtained with one molecule in the asu and was refined to 1.80 Å, but no density for the cofactor was observed in the omit maps. Analysis of the wild‐type structure using PISA^[33]^ supports the experimental data in suggesting a dimer as the majority oligomeric species in solution (Figure 6A). The monomer of ThaADH (Figure 6B) was analyzed using the DALI server^[34]^ and found to have most structural similarity to the alcohol dehydrogenases from *Elizabethkingia anophelis* (PDB 6N7L; 28 % sequence identity; rmsd 1.9 Å over 329 Cα atoms) and *Geobacillus stearothermophilus* (PDB 6IQD;^[35]^ 26 % sequence identity; rmsd 1.7 Å over 327 Cα atoms). While most tertiary structure is conserved between these enzymes, ThaADH has an extended loop between E236 and W249, compared to the equivalent loop V232‐H236 in 6N7L.

The overall structural fold of ThaADH was consistent with those of many other MDR−ADHs,^[36]^ *i. e*. contains the typical C‐terminal coenzyme binding domain and N‐terminal catalytic domain. The domains are separated by a deep cleft containing the active site and offer space for the nicotinamide moiety of the coenzyme and substrate molecule. The remarkable stability of ThaADH in comparison to other MDRs from mesophilic organisms might be due to the different dimer interface. Analysis using PISA^[33]^ suggested that the dimer interface of ThaADH has a salt bridge more than in CPCR2 (PDB 4 C4O)^[37]^ as a less stable related ADH. Furthermore, the dimer interface of ThaADH is more extensive with a corresponding lower Δ^i^G (1778 Å^2^, −23.9 kJ mol^−1^) than in CPCR2 (1484 Å^2^, −19.6 kJ mol^−1^).

ThaADH shares with many ADHs of the MDR family the presence of two zinc atoms per monomer. We confirmed this by atomic absorption spectroscopy (AAS) (Table S2). Four cysteine residues coordinate the structural zinc in the catalytic domain: C94, C97, C100 and C108. However, the description of the coordination of the catalytic zinc located in the active site is more complex. Initially, it was assumed that the catalytic zinc in MDR−ADHs is held exclusively within a tetrahedral coordination structure of three conserved amino acid residues and a water molecule and thus is extremely stationary.^[38]^ Later it was shown that, in response to substrate binding, the position of the catalytic zinc could be altered and zinc ligands exchanged, with penta‐coordinated zinc ion intermediates occurring.^[39]^ The observations of Baker and co‐workers could be substantiated, whereby in the case of the crystal structure of CPCR2 even two different positions of the catalytic zinc could be detected. The distance between the differently positioned catalytic zinc atoms within the same active site amounted to 2.3 Å.^[37]^ Residues coordinating the catalytic zinc in ThaADH are C42, H65, D156 and E66, but superposing ThaADH and CPCR2 structure suggests that the catalytic zinc of ThaADH is in a resting state as already described for CPCR2.^[40]^ In more detail, the distances between the zinc atom and the C4 atom of the nicotinamide ring of the NADH cofactor and the side chain of the residue S46 (CPCR2) and T44 (ThaADH) involved in the proton transfer were compared (Table 3).

**Table 3:** Distances between catalytic zinc of CPCR2 and ThaADH and C4 atom of nicotinamide ring of NADH or the side chain of the amino acid, which is involved in the proton relay, respectively.

Distance between	ThaADH	CPCR2 Zn position A	CPCR2 Zn position B
Zn and NADH	6.0	4.2	6.3
Zn and S46/T44	4.6	4.0	5.6

For reliable docking studies the catalytic zinc needs to be in place in an advantageous position for catalysis.^[41]^ For this reason, molecular docking of substrate molecules into the active site of ThaADH was omitted in this study, and 1,2‐cyclohexanedione was placed manually in the active site. To approximate a productive substrate binding, the maximum distance between the C4 atom of the nicotinamide ring of NADH and the carbonyl carbon of the carbonyl function (<3.2 Å)^[42]^ as well as the distance between the terminal carbonyl oxygen of substrate and the side chain of T44 as proton donor (<3 Å)^[41]^ were taken into account. Thus 1,2‐cyclohexanedione was placed in the active site of ThaADH with 3.0 Å and 2.7 Å distance between the atoms mentioned above, respectively. However, due to the manual placement of the substrate molecule without considering the catalytic zinc, the following models should be treated with caution.

**Figure 7:**
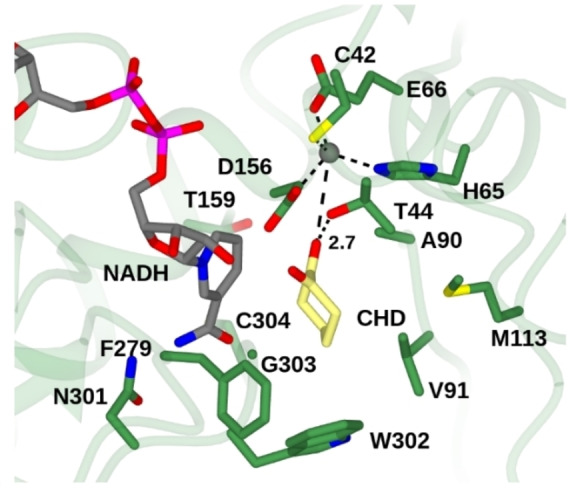
Active site of ThaADH with 1,2‐cyclohexanedione (CHD) modelled into the active site; carbon atoms of side‐chains, NADH and CHD in green, grey and yellow, respectively; selected interactions are indicated by black dashed lines; distances are given in Ångstroms.

Based on the substrate position, the ThaADH substrate binding pocket was examined in a 10 Å radius for residues crucial for substrate binding. Fifteen amino acids were identified that, in addition to participating in catalytic zinc coordination or proton transfer, are responsible for defining the first shell within the active site of ThaADH (Table 4, Figure 7).

**Table 4:** Residues suggested as first shell of the ThaADH active site.

No.	Position	Residue	Function
1	42	Cys	zinc binding
2	44	Thr	proton transfer
3	65	His	zinc binding
4	66	Glu	zinc binding
5	90	Ala	substrate binding
6	91	Val	substrate binding
7	113	Met	substrate binding
8	156	Asp	zinc binding
9	159	Thr	substrate binding
10	279	Phe	substrate binding
11	301	Asn	substrate binding
12	302	Trp	substrate binding
13	303	Gly	substrate binding
14	304	Cys	substrate binding
15	309	Tyr	substrate binding

Superposing the monomeric crystal structure of CPCR2 on ThaADH for comparing the two hydrophobic substrate binding pockets of CPCR2 with the corresponding area of the ThaADH, clearly shows that the active site of ThaADH offers enough space for substrates of the acetophenone type, which we were not able to reduce in this study. Actually, the ThaADH binding pocket generally gapes wider than that of CPCR2 and is more limiting in only a single area, which is due to the position of W302 (Figure 7). In our previous work, we noted the absence of a bulky aromatic residue in the active site of ThaADH,^[19]^ which causes formation of a single large binding pocket in contrast to the two‐pocket model of reference ADHs like CPCR2.^[9]^ Hence, the shape of the substrate‐binding pocket of ThaADH is not evolved for a catalytically active positioning of substrates with a small and large substituent at the carbonyl function such as acetophenone.

**Figure 8:**
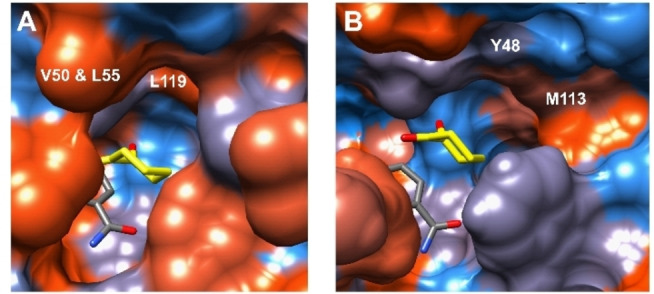
Active site surface of CPCR2 (A) and ThaADH (B) with 1,2‐cyclohexanedione modelled into the substrate binding pocket. The most hydrophobic amino acids are colored orange, more hydrophilic residues are colored blue.

In fact, the superposition of both crystal structures shows, that the restricting W286 of CPCR2 is exchanged for the small G303 of ThaADH, complicating a clear distinction between the otherwise existing two substrate binding pockets. Another structural difference could also have an impact on the variation of the substrate specificity of ThaADH and CPCR2: In the large substrate binding pocket of CPCR2 three hydrophobic residues were found: V50, L55 and L199. In contrast, the corresponding ThaADH residues Y48 and M113 are much more hydrophilic (Figure 8A & B). A more hydrophilic binding pocket may cause the rejection of aromatic substrates like acetophenone, because of the lack of or the reduction of hydrophobic interactions.

**Figure 9:**
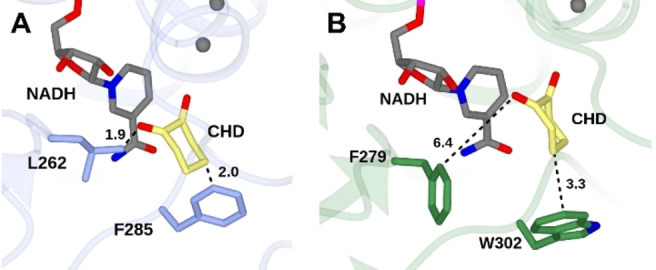
Active site of CPCR2 (A) and ThaADH (B) with 1,2‐cyclohexanedione modelled into the substrate binding pocket. Backbone carbon atoms for CPCR2 and ThaADH are shown in blue and green, respectively. Crucial residues for substrate binding are highlighted. Black dashed lines show selected distances in Ångstroms.

Manual placement of 1,2‐cyclohexanedione in the active site of CPCR2 as described above leads to potential clashes of the molecular surface between the substrate and two residues: F285 in 2.0 Å distance to the C4 and L262 in 1.9 Å distance to the second carbonyl function of 1,2‐cyclohexanedione, respectively (Figure 9A).

This is in good agreement with the experimental finding that CPCR2 is not able to convert the cyclic diketone.^[19]^ With W302, ThaADH also has a large aromatic amino acid corresponding to F285 of CPCR2. But due to its slightly offset orientation, W302 frees up more space around the C4 of 1,2‐cyclohexanedione (3.3 Å) in the active site of ThaADH. Nevertheless, W302 could prevent the productive substrate binding of sterically demanding ɣ‐substituted carbonyl compounds by ThaADH. Regarding L262 of CPCR2, the ThaADH also has the bulky residue F279 at the corresponding position, but this does not limit the space around the second carbonyl function of the substrate (6.4 Å) again due to an offset orientation (Figure 9B). In contrast to previous assumptions,^[19]^ the already mentioned W286 of CPCR2 seems not to be critical for substrate binding, but instead F285 and L262.

## Conclusion

The zinc‐dependent ThaADH represents a MDR−ADH with a unique substrate preference for sterically demanding α‐substituted linear cyclic carbonyl compounds, and a high substrate specificity. It also has a comparatively high thermal, pH‐ and solvent stability, and accepts the alternative cofactor cNAD^+^_._ Thus, it combines various promising features for applied biocatalysis. It accepts a limited, but potentially interesting substrate range for both oxidation and reduction. Resolving the structure of WT ThaADH and a double mutant and comparing this to related well‐studied MDR−ADHs with preference for phenyl substituted and linear aliphatic substrates, gave novel insights into the underlying structure‐function relationships, which in the future might enable the rational design of MDR−ADHs towards acceptance of a wider range of diketones and their corresponding alcohols. Nevertheless, further investigation might be required as our previous studies indicate that refinement of single structural determinants will not be sufficient.^[19]^ Identification of functionally more related ADHs would also help to untangle the contribution and relation of structural determinants for substrate specificity further.

## Experimental Section

**Chemicals**: Unless stated otherwise, all chemicals, media and enzymes were purchased from Sigma‐Aldrich Merck (St. Louis, MO, USA), AppliChem GmbH (Darmstadt, Germany) and Carl Roth GmbH & Co. (Karlsruhe, Germany).

**Expression of** ***thaADH***: For the production of WT ThaADH either with C‐terminal Strep‐tag or N‐terminal hexahistidine‐tag (His‐tag) and its K319A/K320A double mutant, *E. coli* BL21 (DE3) cells were first transformed with a pET22b containing the relevant genes. The ThaADH gene (Genbank GI: 19571180) was achieved from genomic DNA from *Thauera aromatica* K172 (DSM6984). The double mutation was predicted for facilitating the crystallization by the surface entropy reduction prediction server (SERp server). Expression was performed in 1 L Luria‐Bertani medium containing either 100 mg mL^−1^ kanamycin (His‐tagged ThaADH WT and mutant) or 200 mg mL^−1^ ampicillin (Strep‐tagged ThaADH) as an antibiotic marker. Medium was inoculated from an overnight culture to a final OD_600_ of 0.02 and incubated at 37 °C with shaking at 180–220 r.p.m. At an OD_600_ of 0.7–1.0, expression was induced by the addition of 1 mmol L^−1^ (His‐tagged ThaADH and mutant) or 2.5 mmol L^−1^ (Strep‐tagged ThaADH) IPTG and the temperature was reduced to 20 °C. In addition, 0.43 mmol L^−1^ ZnSO_4_ was added for the production of active zinc‐containing alcohol dehydrogenases as reported for SsADH from *Sulfolobus solfataricus* ^[43]^ and ADH−T from *Thermoanaerobacter* sp.^[14]^ The cells were harvested after 21 h (His‐tagged ThaADH WT and mutant) or 46 h (Strep‐tagged ThaADH) by centrifugation (4 °C, 5000 rpm, 30 min) and stored at −20 °C (8000 rpm, 15 min, 4 °C).

**Purification of Strep‐tagged ThaADH**: Cells were resuspended in cell lysis buffer with pH 8.0 containing 100 mmol L^−1^ triethanolamine (TEA), 150 mmol L^−1^ NaCl, 5 mmol L^−1^ MgCl_2_, 1 mmol L^−1^ phenylmethylsulfonyl fluoride (PMSF) and 50 mg L^−1^ DNaseI. The disruption of cells was performed by two passages of French press treatment at 1200 Psi. Cell debris was removed by centrifugation (14000 rpm, 1 h, 4 °C). Crude extract containing the ThaADH‐Strep‐tag fusion protein was applied to affinity chromatography, using a 5 mL Strep‐Tactin Superflow high capacity column (IBA GmbH, Göttingen, Germany) according to the manufacturer‘s instruction with the following buffers: washing buffer (100 mmol L^−1^ TEA, 150 mmol L^−1^ NaCl at pH 7.5), elution buffer (100 mmol L^−1^ TEA, 150 mmol L^−1^ NaCl, 2.5 mmol L^−1^ desthiobiotin at pH 7.5). ThaADH concentrations were determined by a bicinchoninic acid assay kit (Thermo Fisher Scientific, Waltham, MA, USA) according to the manufacturer‘s instruction. Bovine serum albumin was used as standard. Fractions showing high enzyme activity were pooled and analyzed by SDS‐PAGE. Strep‐tagged ThaADH was stored at 6 °C and was used for all studies apart from protein crystallization.

**Purification of His‐tagged ThaADH and K319A/K320A mutant**: Cell pellet were resuspended in washing buffer, pH 7.5, containing 20 mmol L^−1^ potassium phosphate buffer, 500 mmol L^−1^ NaCl, 20 mmol L^−1^ imidazole, 10 % (v/v) glycerol and 0.1 % (v/v) Tween 20. Cell lysis was carried out by two passages through a French pressure cell at 26 kPsi. The cell lysate was centrifuged (14000 rpm, 1 h, 4 °C). Crude extract containing the His‐tag‐ThaADH fusion protein was loaded onto a 5 mL HisTrap HP column for a nickel‐nitrilotriacetic acid based protein purification. The column was washed with washing buffer and the enzyme was eluted by increasing the imidazole concentration with a linear gradient up to 300 mmol L^−1^ imidazole. Peak fractions were first analyzed by SDS‐PAGE and then pooled and concentrated to load 2‐mL samples onto a pre‐equilibrated HiLoad Superdex column for a preparative SEC. Enzyme was eluted by using the SEC buffer (20 mmol L^−1^ potassium phosphate buffer pH 7.5, 500 mmol L^−1^ NaCl). Selected fractions containing the biocatalyst were analyzed by SDS‐PAGE. Fractions containing the biocatalyst were pooled and concentrated for crystallization trials.

**Analytical size exclusion chromatography**: For the investigation of the oligomerization state of the native enzyme a HiLoad 16/60 Superdex 75 prep grade filtration column was used for size exclusion chromatography. The column was equilibrated with running buffer (100 mmol L^−1^ TEA pH 7.5, 150 mmol L^−1^ NaCl) before sample loading (1 mg ThaADH; 1–2.5 mg standard protein, respectively) and elution. For calibration (Figure S4) the Gel Filtration HMW Calibration Kit (GE Healthcare, Chalfont Saint Giles, UK), CPCR2 dimer[^31^, ^37^] and Lipase B from *Candida antarctica* ^[44]^ were used. Calculation of K_av_ values was done with the Equation 1:

(1) $$K_{av}=\frac{V_{e}-V_{0}}{V_{t}-V_{0}}$$

K_av_=elution volume parameter; V_e_=elution volume for the protein; V_t_=total bed volume; V_0_=column void volume.

**Blue native PAGE**: Blue native PAGE with gradient gels (3–12 %) and gel staining was conducted according to the manufacturer's instructions of the SERVAGel N Native Gel Starter Kit (Serva Electrophoresis GmbH, Heidelberg, Germany). The SERVA Native Marker Liquid Mix and 20 μg ThaADH was loaded onto the gels.

**ThaADH activity assay**: Initial activities were determined photometrically using a Cary 60 UV‐vis spectrophotometer (Agilent, Waldbronn, Germany) by measuring the consumption of the cofactor NADH (reduction) and NAD^+^ (oxidation), respectively. Absorption at 340 nm was measured for 2 min at 40 °C in triplicate. The standard reduction assay was performed in 100 mmol L^−1^ potassium phosphate buffer at pH 6.5, 25 mmol L^−1^ substrate, 0.25 mmol L^−1^ NADH and an appropriate concentration of ThaADH in 1 mL scale. The oxidation assay was carried out in 100 mmol L^−1^ HEPES buffer at pH 9.0, 25 mmol L^−1^ substrate, 1 mmol L^−1^ NAD^+^ with adequate concentrations of ThaADH in 1 mL scale. For the investigation of the substrate range, the substrate concentration was 10 mmol L^−1^. For thermal stability studies, the enzyme was incubated in 100 mmol L^−1^ TEA, 150 mmol L^−1^ NaCl, pH 7.5 at the specific temperature for various lengths. For the investigation of pH‐dependent stability, the enzyme was incubated at room temperature in 100 mmol L^−1^ buffer with appropriate pH for various lengths. Afterwards the reductive activity was determined as described above. In case of the investigation of the pH‐dependency of ThaADH activity, the described activity assay was varied in the type of buffer and pH. The half‐life times were calculated by the inactivation constant, which is the slope of the natural logarithm of the activity as a function of the incubation time.

(2) $$t_{1/2}=\;\frac{ln\;2}{k}$$

t_1/2_=half‐life; k=inactivation constant

To verify the effect of organic solvents on the ThaADH activity, each solvent was added to the assay in different concentrations. For the investigation of the solvent‐dependent stability, the enzyme was incubated in 20 % (v/v) polar organic solvent. Afterwards the reductive activity was determined as described above. For kinetic studies, the substrate concentrations of the photometric standard assay were varied.

**Biotransformation for kinetics and product identification**: An enzyme‐coupled cofactor regeneration system with the RjFDH from *Rhodococcus jostii* ^[45]^ was used for the preparative biotransformation in a 5 mL (kinetics) or 50 mL batch (product identification) at room temperature. The reaction was performed in 0.1 mol L^−1^ potassium phosphate buffer at pH 6.5, 0.25 mmol L^−1^ NADH, 2 mmol L^−1^ to 120 mmol L^−1^ substrate, if necessary up to 5 % (v/v) DMSO. The FDH activity was used in a three‐fold excess over the ADH activity; likewise the FDH substrate formate was in excess over the ADH substrate.

**Product analytics**: The extraction of substrates and products was performed with equal volumes of ethyl acetate. To monitor the reduction reaction a Shimadzu GC‐2010 chromatograph system with flame ionization detector and nitrogen as carrier gas was used. For products of diketone reduction a HP‐Chiral‐20B column with 30 m length and 0.25 mm inner diameter (Agilent Technologies, Santa Clara, USA) and for products of the halogenated cyclohexanones reduction a Hydrodex γ‐DiMOM column with 25 m length and 0.25 mm inner diameter (Macherey‐Nagel, Düren, Germany) were employed. For analysis, the following temperature programs were applied: 90–120 °C with ramp of 2.5 °C min^−1^ and 120–220 °C with ramp of 15 °C min^−1^ (1,2‐cyclohexanedione), 100–115 °C with ramp of 0.75 °C min^−1^ (2‐bromocyclohexanone), 100–125 °C with ramp of 2.5 °C min^−1^ (2‐chlorocyclohexanone) and 75–88 °C with ramp of 0.75 °C min^−1^ (2‐fluorocyclohexanone). Retention times of substrate 1,2‐cyclohexanedione and product 2‐hydroxycyclohexanone were 14.8 min, 15.2 min ((*R*)‐product) and 15.4 min ((*S*)‐product), respectively. The (*S*)‐substrates of racemic halogenated cyclohexanones were observed at 17.6 min (2‐bromocyclohexanone), 8.5 min (2‐chlorocyclohexanone) and 13.4 min (2‐fluorocyclohexanone), respectively; (*R*)‐substrates were detected at 18.5 min (2‐bromocyclohexanone), 8.8 min (2‐chlorocyclohexanone) and 14.3 min (2‐fluorocyclohexanone), respectively. These programs could separate the four possible products of the reduction only partially. Product peaks were monitored at 17.1 min (2‐bromocyclohexanol), 7.8 min (2‐chlorocyclohexanol), 8.5 min and 9.5 min (2‐fluorocyclohexanonl), respectively. For the separation of reaction products and remaining substrates the reaction mixture was subjected to a liquid chromatography on silica gel. Afterwards an Agilent 6890N GC/5973N Q MS system equipped with an Optima 5 HT column with 30 m length and 0.25 mm inner diameter (Macherey‐Nagel, Düren, Germany) was used for substance identification. For the product analysis the following temperature programs were used: 35–300 °C with ramp of 10 °C min^−1^ (1,2‐cyclohexanedione), 70–320 °C with ramp of 10 °C min^−1^ (2‐bromocyclohexanone and 2‐chlorocyclohexanone). Retention times of 2‐hydroxycyclohexanone, 2‐bromocyclohexanol and 2‐chlorocyclohexanol were 12.3 min, 7.3 min 5.9 min, respectively. ^1^H‐NMR and ^13^C‐NMR spectroscopy were used to analyze the reaction products and remaining substrates (400 MHz, Bruker DRX‐500 P) and to distinguish between the product diastereomers of the halogenated cyclohexanone reduction (600 MHz, Bruker AV III 600). For this the purified substance was solved in chloroform‐d (^1^H: δ=7.26, ^13^C: δ=77). To differentiate between enantiomers and diastereomers of substrates and products, the absolute configurations of substances were determined by a Perkin Elmer 341 LC polarimeter. Chloroform and benzene were used as solvent. Specific rotation values were compared to literature.

**Atomic absorption spectroscopy**: Zinc level per subunit was analyzed using a Carl Zeiss atomic absorption spectrophotometer AAS5 at 214 nm. For this 4 mg ThaADH in 100 mmol L^−1^ TEA buffer pH 7.5 were used.

**Protein crystallization**: Pure His‐tagged WT ThaADH and its K319A/K320A mutant at a concentration of 25 mg mL^−1^ and 50 mg mL^−1^ in complex with 10 mmol L^−1^ NADH was subjected to crystallization trials using a range of commercially available screens in 96‐well plates using 300 nL drops with 150 nL protein solution and 150 nL of precipitant solution. The best hits were obtained in conditions containing 0.1 mol L^−1^ Tris buffer pH 8.5, 0.05 mol L^−1^ MgCl_2_ and 12 % (v/v) methylpentanediol (MPD). For the K319A/K320A mutant best crystals were obtained in 0.1 mol L^−1^ bis‐Tris buffer, pH 6.5 with 25 % (w/v) PEG‐3350. Crystals were flash‐cooled in liquid nitrogen without cryoprotectant in the case of the WT, but with the mother liquor plus 10 % (v/v) ethylene glycol for the mutant, and tested for diffraction using a Rigaku Micromax‐007HF fitted with Osmic multilayer optics and a MARRESEARCH MAR345 imaging plate detector. Crystals that displayed diffraction of better than 3 Å resolution were retained for data collection at the synchrotron.

**Data collection and processing, structure solution and refinement**: Datasets for crystals of both WT ThaADH in complex with NAD^+^ and the K319A/K320A apo‐mutant were collected on beamline I04‐1 at the Diamond Light Source Synchrotron in Oxford, UK. Data, which were collected to 2.60 and 1.80 Å, respectively, were processed and integrated using XDS^[46]^ and scaled using SCALA^[47]^ as part of the Xia2 processing system.^[48]^ Data collection statistics are given in Table 5. The structures were solved with the program MOLREP,^[49]^ using a monomer of the D‐arabinose dehydrogenase from *Sulfolobus solfataricus* (PDB 2H6E)^[50]^ as a model. The solutions of WT and K319A/K320A contained three and one molecule in the asymmetric unit, respectively. The structure was built and refined using iterative cycles within the programs COOT^[51]^ and REFMAC,^[52]^ respectively. After building the protein and water molecules in the WT structure, clear density was observed for the cofactor NAD^+^ in all three monomers. The WT structure was refined to R_cryst_ and R_free_ values of 21.7 % and 25.3 %, respectively. The K319A/K320A mutant, in which no density for NAD^+^ was visible in the omit maps, was refined to R_cryst_ and R_free_ values of 21.2 and 27.4 %, respectively. Each structure was validated upon deposition within the Protein DataBank (PDB). Refinement statistics are presented in Table 5. Coordinates for the ThaADH WT−NAD^+^ complex and K319A/K320A mutant structures have been deposited in the PDB with the accession codes 7QUY and 7QUL, respectively.

**Table 5:** Data collection and refinement statistics for ThaADH K319A/K320A apo and ThaADH WT in complex with NAD^+^. Numbers in brackets refer to data for highest resolution shells.

	ThaADH K319A/K320A (apo)	ThaADH WT (NADH)
Beamline	Diamond I04‐1	Diamond I04‐1
Wavelength (Å)	0.915890	0.915890
Resolution (Å)	36.45–1.80 (1.84–1.80)	97.56–2.60 (2.69–2.60)
Space group	*I*2_1_	*C*222_1_
Unit cell (Å)	a=51.62; b=72.89; c=81.28 α=γ=90.00°; β=103.59	a=75.42; b=239.33; c=168.46 α= β=γ=90.00°
No. of molecules in the asu	1	3
Unique reflections	27158 (1606)	47369 (4598)
Completeness (%)	99.9 (100.0)	100.0 (99.9)
R_merge_ (%)	0.13 (0.37)	0.09 (1.11)
R_p.i.m._	0.11 (0.32)	0.05 (0.63)
Multiplicity	4.0 (3.7)	8.1 (7.8)
<*I*/*σ(I)*>	6.1 (3.4)	15.6 (1.8)
Overall B factor from Wilson plot (Å^2^)	14	58
CC_1/2_	0.97 (0.62)	1.00 (0.79)
R_cryst_/R_free_ (%)	21.2/27.4	21.7/25.3
r.m.s.d 1–2 bonds (Å)	0.009	0.010
r.m.s.d 1–3 angles (°)	1.64	1.98
Avge main chain B (Å^2^)	20	71
Avge side chain B (Å^2^)	22	75
Avge water B (Å^2^)	30	54
NAD^+^ B (Å^2^)	–	68

## Conflict of interest

The authors declare no conflict of interest.

1

## Supporting information

As a service to our authors and readers, this journal provides supporting information supplied by the authors. Such materials are peer reviewed and may be re‐organized for online delivery, but are not copy‐edited or typeset. Technical support issues arising from supporting information (other than missing files) should be addressed to the authors.

Supporting Information

## Data Availability

The data that support the findings of this study are available from the corresponding author upon reasonable request.
