# Supplementary material for: Advanced Insights into Catalytic and Structural Features of the Zinc‐Dependent Alcohol Dehydrogenase from Thauera aromatica
Source: Chembiochem. 2022 Jun 14;23(15):e202200149. doi: 10.1002/cbic.202200149 (PMC9400901; doi:10.1002/cbic.202200149)
Supplement: Supplementary file 1 — Supporting Information [file CBIC-23-0-s001.pdf]

ChemBioChem

Supporting Information

Advanced Insights into Catalytic and Structural Features of the Zinc-Dependent Alcohol Dehydrogenase from *Thauera aromatica*

Frances Stark, Christoph Loderer, Mark Petchey, Gideon Grogan,* and Marion B. Ansorge-Schumacher*

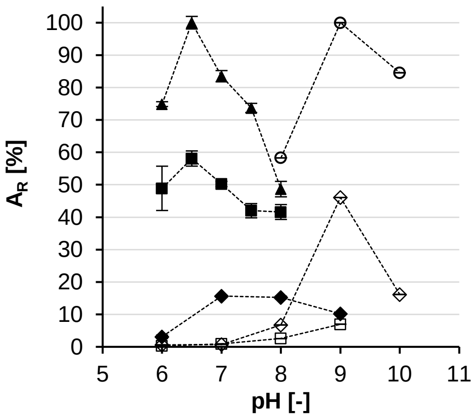

Figure S1. pH-dependent activity of ThaADH in the range of pH 6 to 10. Initial activities were measured according to the standard activity assay of 1,2-cyclohexanedione reduction and 1,2-cyclohexanediol oxidation with various buffers and pH values, respectively. Filled symbols: reduction, open symbols: oxidation, squares: potassium phosphate buffer, circles: HEPES buffer, triangles: PIPES buffer, rhombus: TEA buffer. 100 % relative activity: 69.8 U mg-[1] (reduction) and 0.2 U mg^{-1} (oxidation), respectively. A_R : relative activity

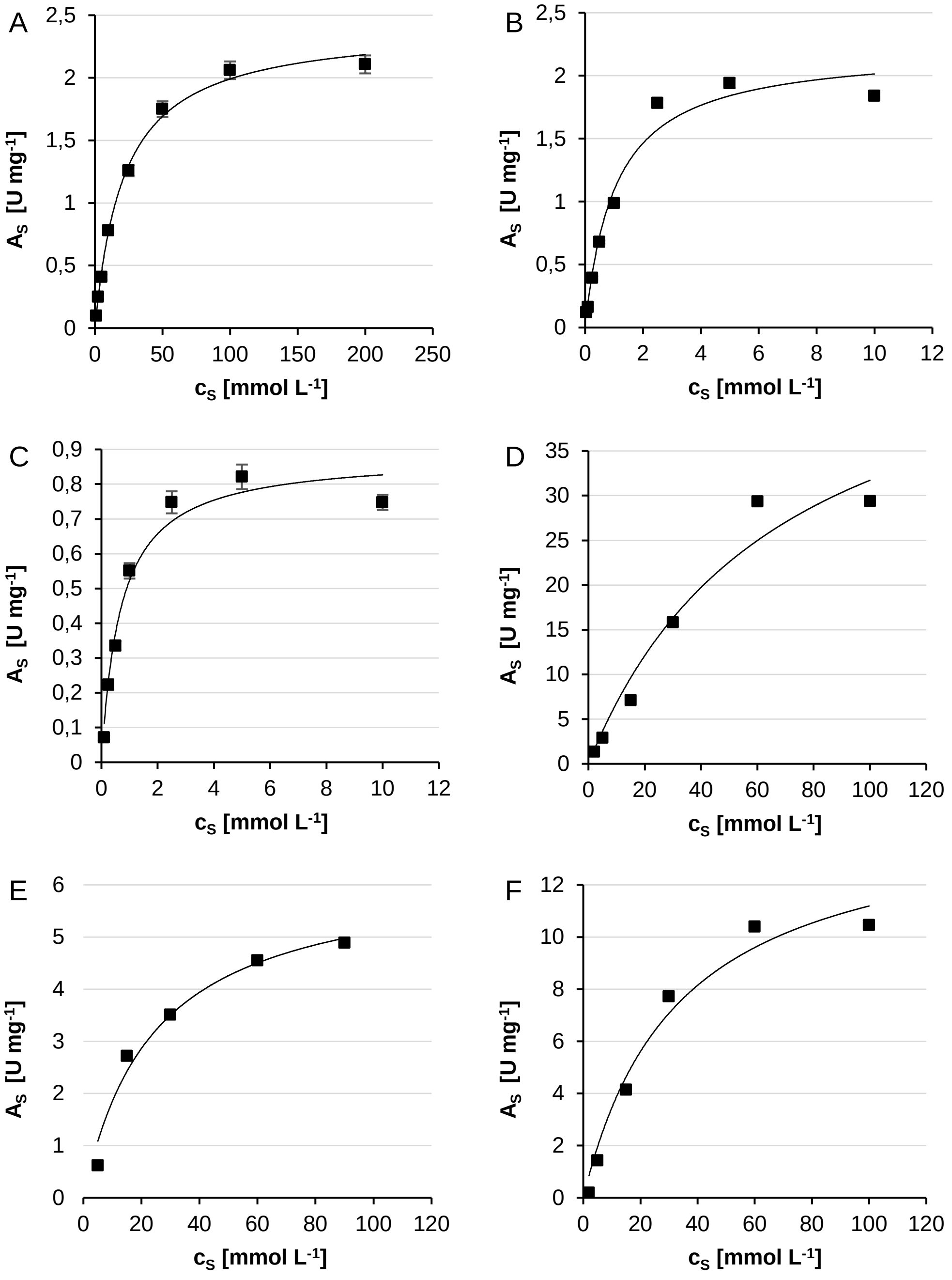

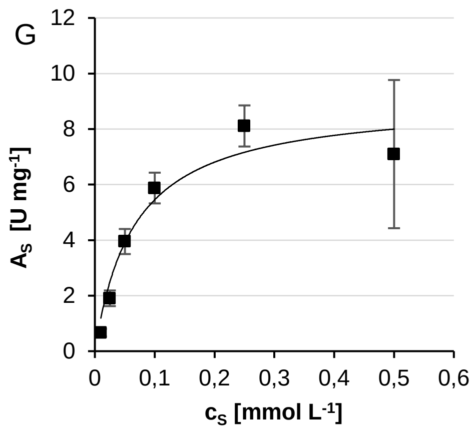

Figure S2. Kinetic data and Michaelis-Menten nonlinear regression fitting of ThaADH catalyzed reduction and oxidation of 3-methylcyclohex-2-en-1-ol (A), NAD^+ (B), $cNAD^+$ (C), 1,2-cyclohexanedione (D), 2-bromocyclohexanone (E), 2-chlorocyclohexanone (F), NADH (G). A_S: specific activity, c_S: substrate concentration

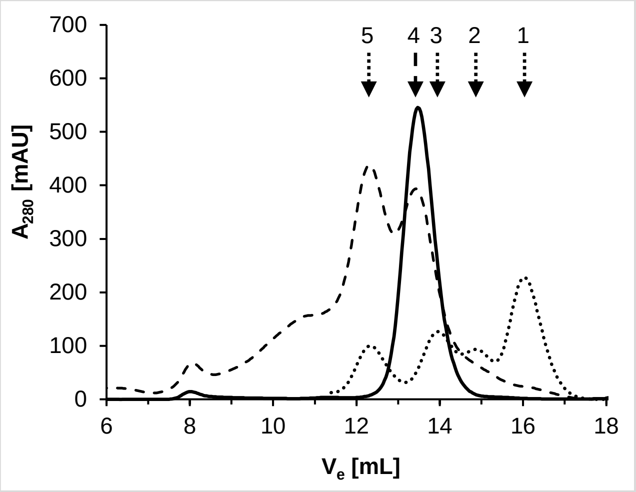

Figure S3. Gel filtration chromatogram for determination of the ThaADH molecular mass and oligomerization state. Elution profiles of ThaADH (solid line), CPCR2 (dashed line) and standard proteins (dotted line) result from the absorption at 280 nm (A_{280}) of fractions with different elution volume (V_e). Proteins for calibration (Figure S4): CalB, 33 kDa (1), ovalbumin, 43 kDa (2), conalbumin, 75 kDa (3), CPCR2 dimer, 78 kDa (4), aldolase 158 kDa (5).

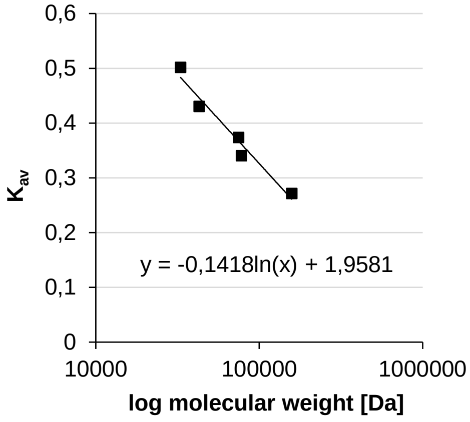

Figure S4. Calibration curve of analytical size exclusion chromatography showing the elution volume parameter (K_{av}) and the logarithmic molecular weight of CalB (33000 Da), ovalbumin (43000 Da), conalbumin (75000 Da), CPCR2 dimer (78000 Da) and aldolase (158000 Da) as standard proteins.

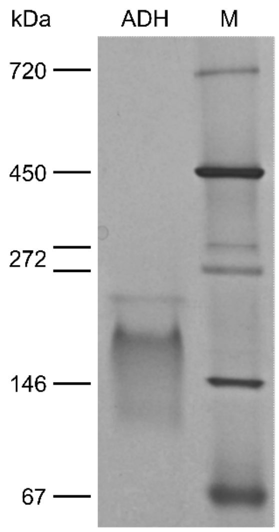

Figure S5: Blue native PAGE for determination of the ThaADH molecular mass and oligomerization state. 20 µg ThaADH (ADH) and Marker (M) were loaded onto the gel.

Table S1. Optical rotatory power of rejected substrates and preferred products of ThaADH from reduction reactions, and enantiomeric/diastereomeric excess of reaction products.

substrate	product	$[\alpha]_D^{20\,°C}$		ee/de [%] of product
		rejected substrate enantiomer	preferred product enantiomer/diastereomers	
1,2-cyclohexanedione	2-hydroxycyclohexanone	-	-26.8 (S) (c 0.65, $CHCl_3$)	66 % (S)
2-bromocyclohexanone	2-bromocyclohexanol	-171.3 (R) (c 0.5, $CHCl_3$)	+25.52 (1S,2S) (c 1, $CHCl_3$)	88 % trans
2-chlorocyclohexanone	2-chlorocyclohexanol	-64.7 (R) (c 0.28, $CHCl_3$)	+24.6 (1S,2S) (c 1, $CHCl_3$)	81 % trans

c: substance concentration [g 100 mL^{-1}], α: specific rotation at 20 °C, 589 nm

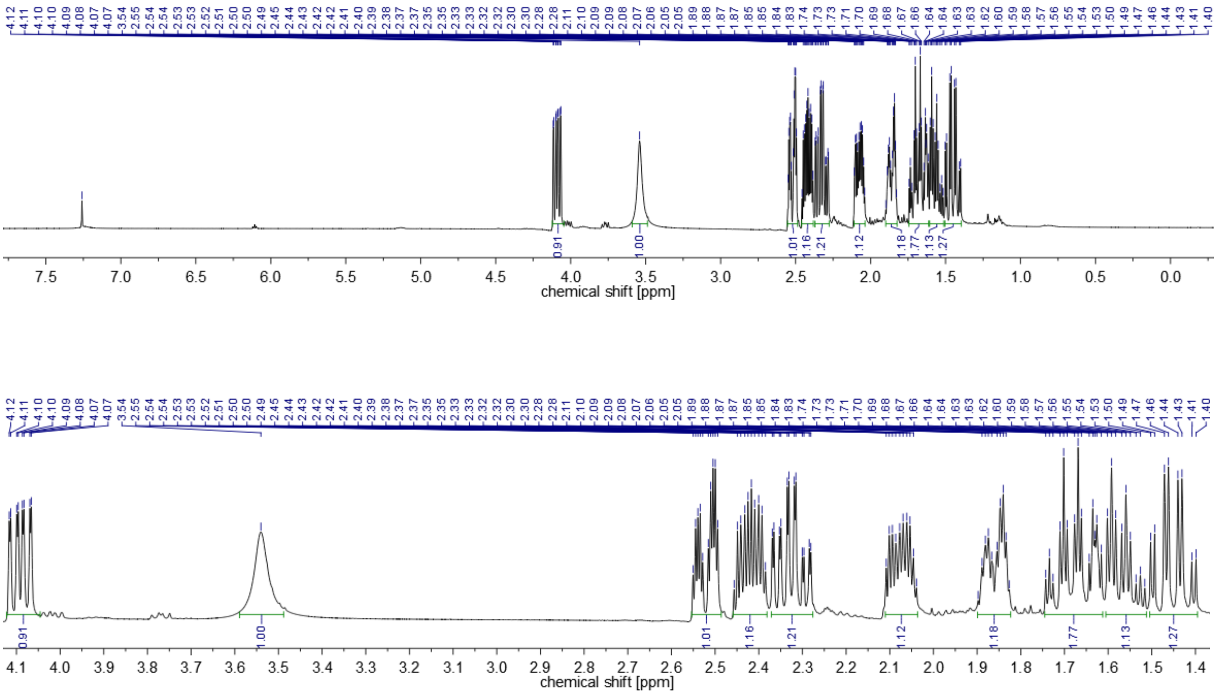

Figure S6. ^{1}H-NMR spectrum in $CDCl_3$ of 2-hydroxycyclohexanone as product of 1,2-cyclohexanedione reduction with ThaADH. The obtained hydroxyl group is represented by the singlet at 3.54 ppm.

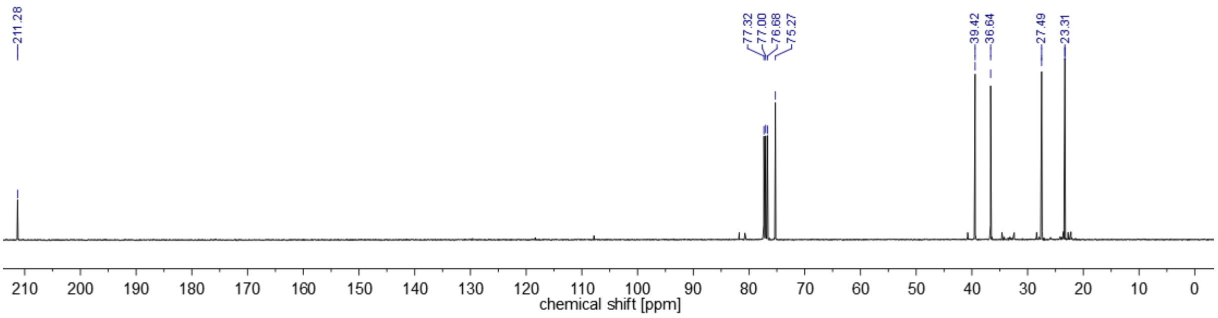

Figure S7. ^{13}C-NMR spectrum in $CDCl_3$ of 2-hydroxycyclohexanone as product of 1,2-cyclohexanedione reduction with ThaADH. The hydroxy group-bearing carbon atom is represented by the signal at 211.28 ppm.

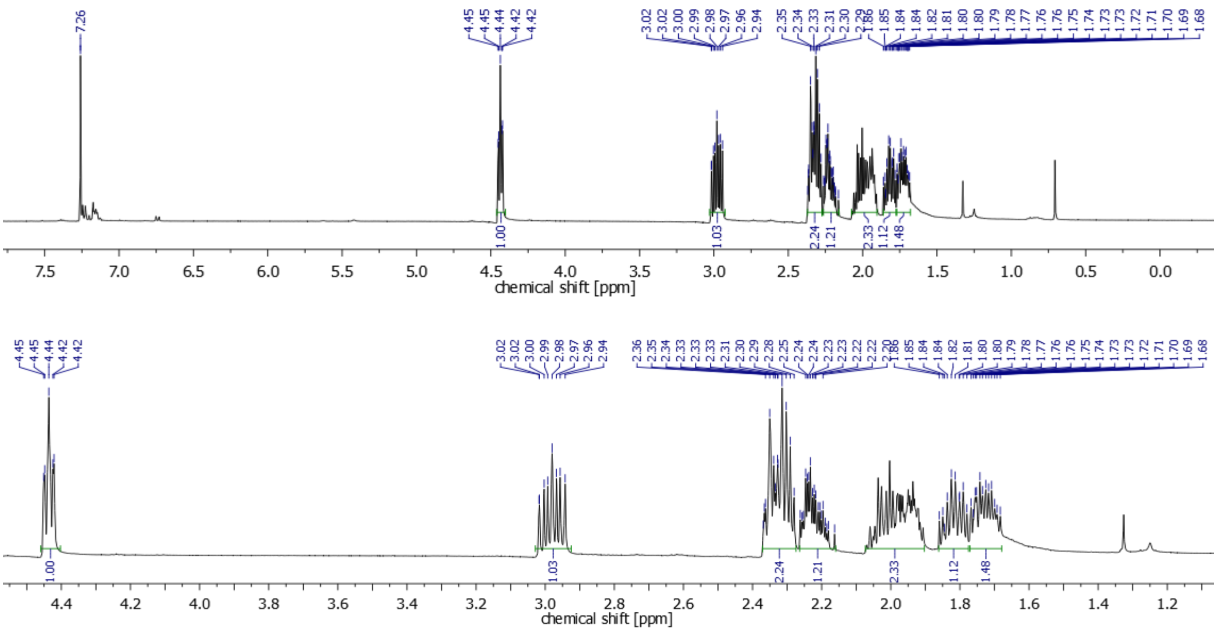

Figure S8. ^{1}H-NMR spectrum in $CDCl_3$ of 2-bromocyclohexanone as rejected substrate of ThaADH catalyzed 2-bromocyclohexanol production.

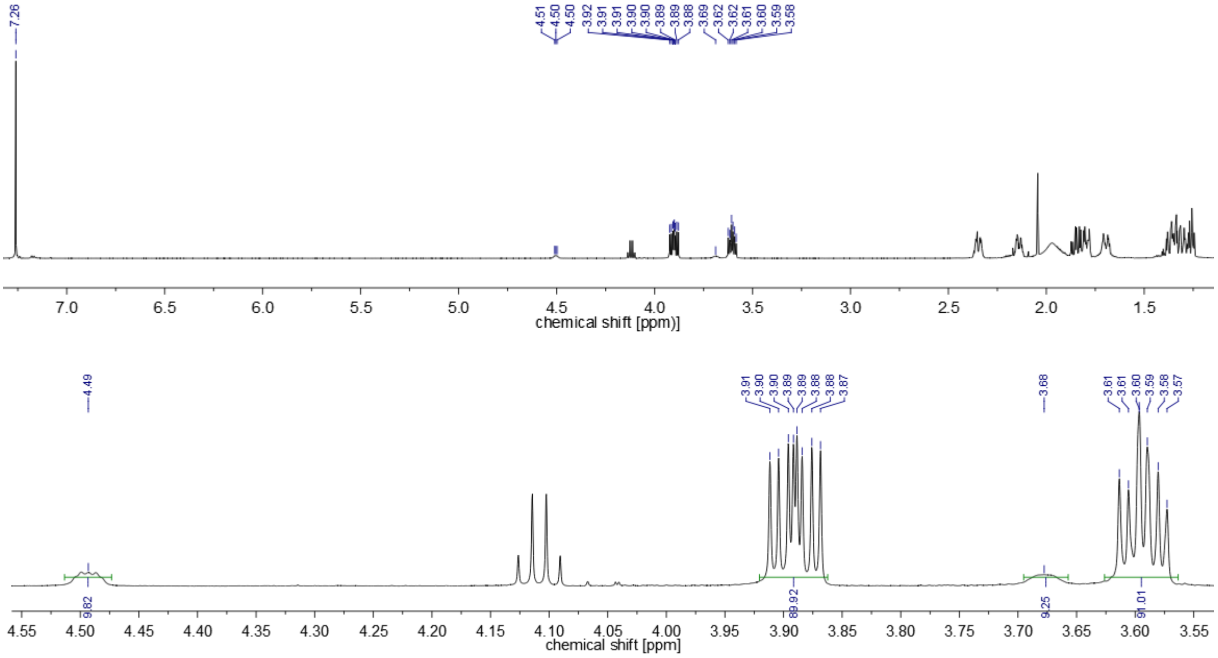

Figure S9. ^{1}H-NMR spectrum in $CDCl_3$ of 2-bromocyclohexanol as preferred product of 2-bromoyclohexanone reduction with ThaADH. Because of multiple proton coupling the hydroxy groups of cis and trans configuration resulting from the biocatalyst activity are represented by the signal at 3.67-3.68/4.49-4.50 ppm and 3.57-3.61/3.87-3.91 ppm, respectively. The ratio of the specified peak areas was used to calculate de.

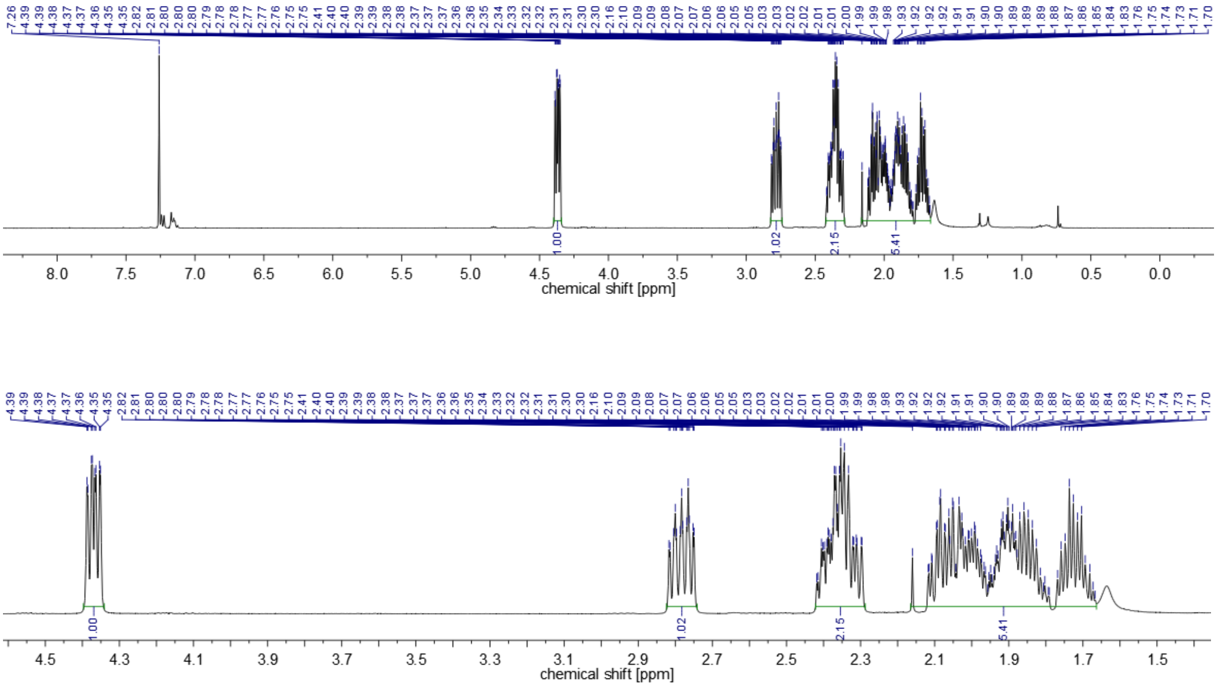

Figure S10. ¹H-NMR spectrum in $CDCl_3$ of 2-chlorocyclohexanone as rejected substrate of 2-chlorocyclohexanol production with ThaADH.

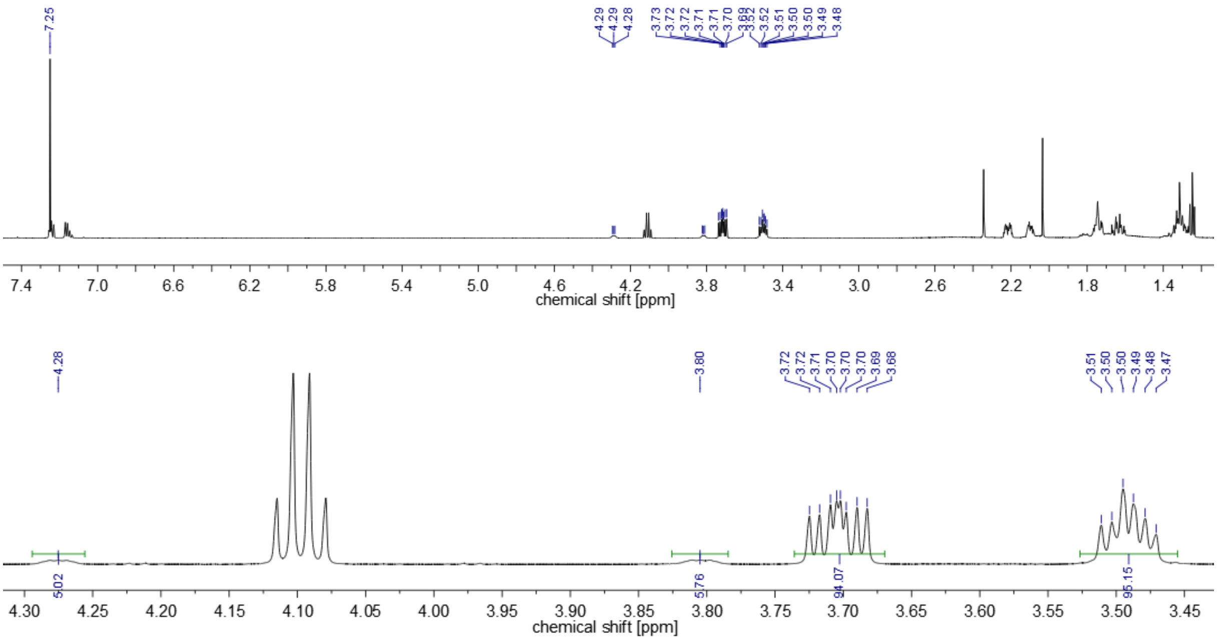

Figure S11: ₁H-NMR spectrum in $CDCl_3$ of 2-chlorocyclohexanol as preferred product of 2-chlorocyclohexanone reduction with ThaADH. Because of multiple proton coupling the hydroxy groups of cis and trans configuration resulting from the biocatalyst activity are represented by the signal at 3.80-3.81/4.27-4.28 ppm and 3.47-3.51/3.68-3.72 ppm, respectively. The ratio of the specified peak areas was used to calculate de.

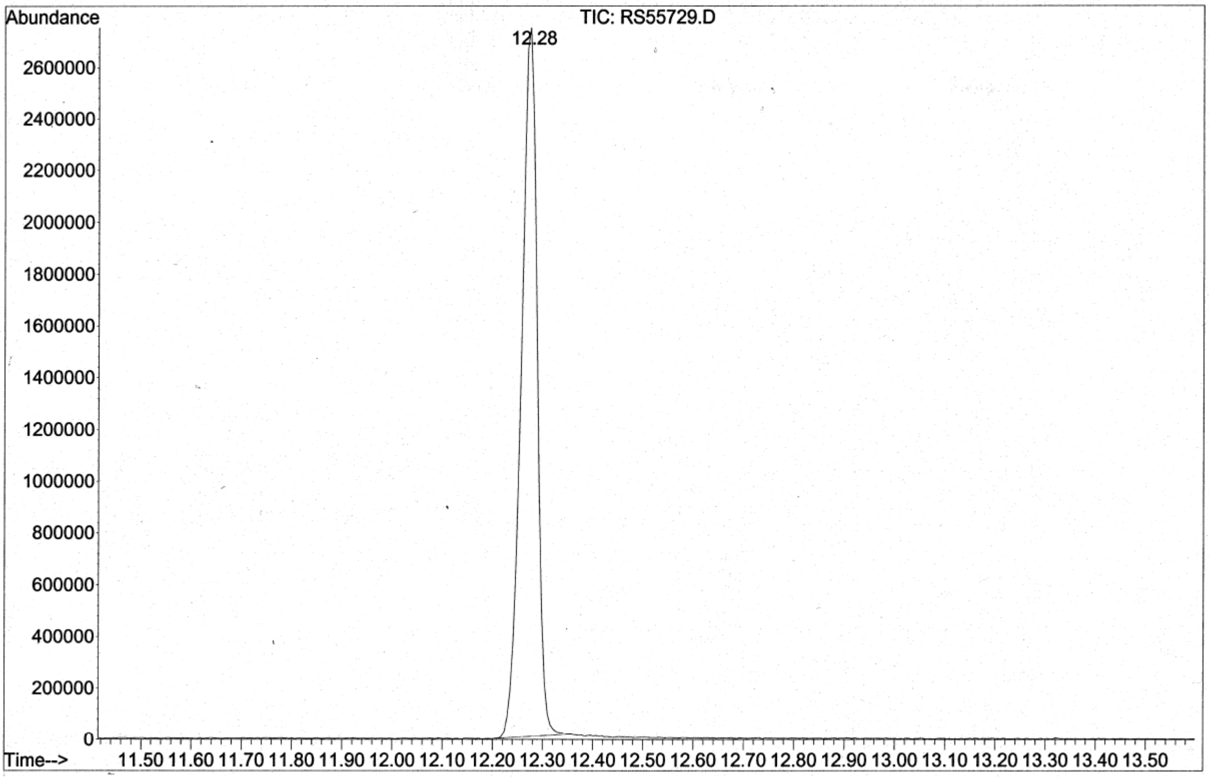

Figure S11. Chromatogram of the GC-MS analysis of purified 2-hydroxycyclohexanone as product of 1,2-cyclohexanedione reduction with ThaADH. Peak at 12.28 min

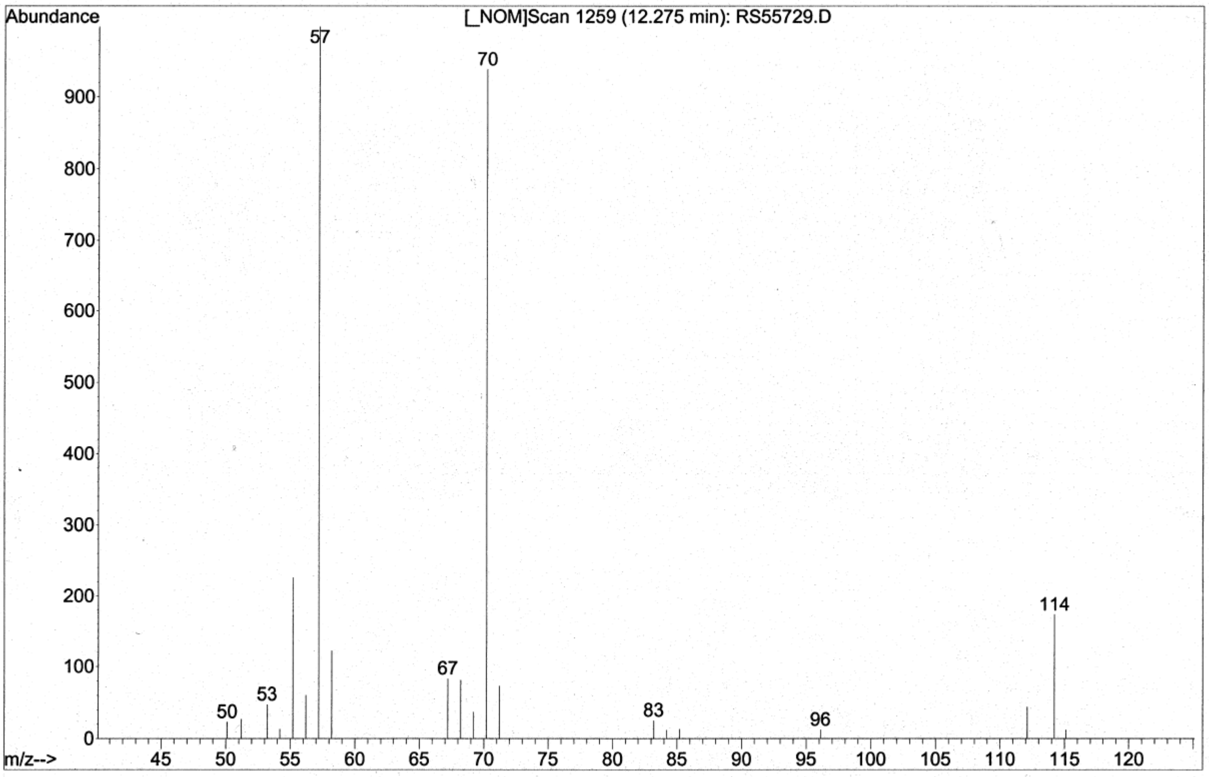

Figure S12. Mass spectrum of purified 2-hydroxycyclohexanone as product of 1,2-cyclohexanedione reduction with ThaADH. Compound was eluted at 12.28 min (Figure S12).

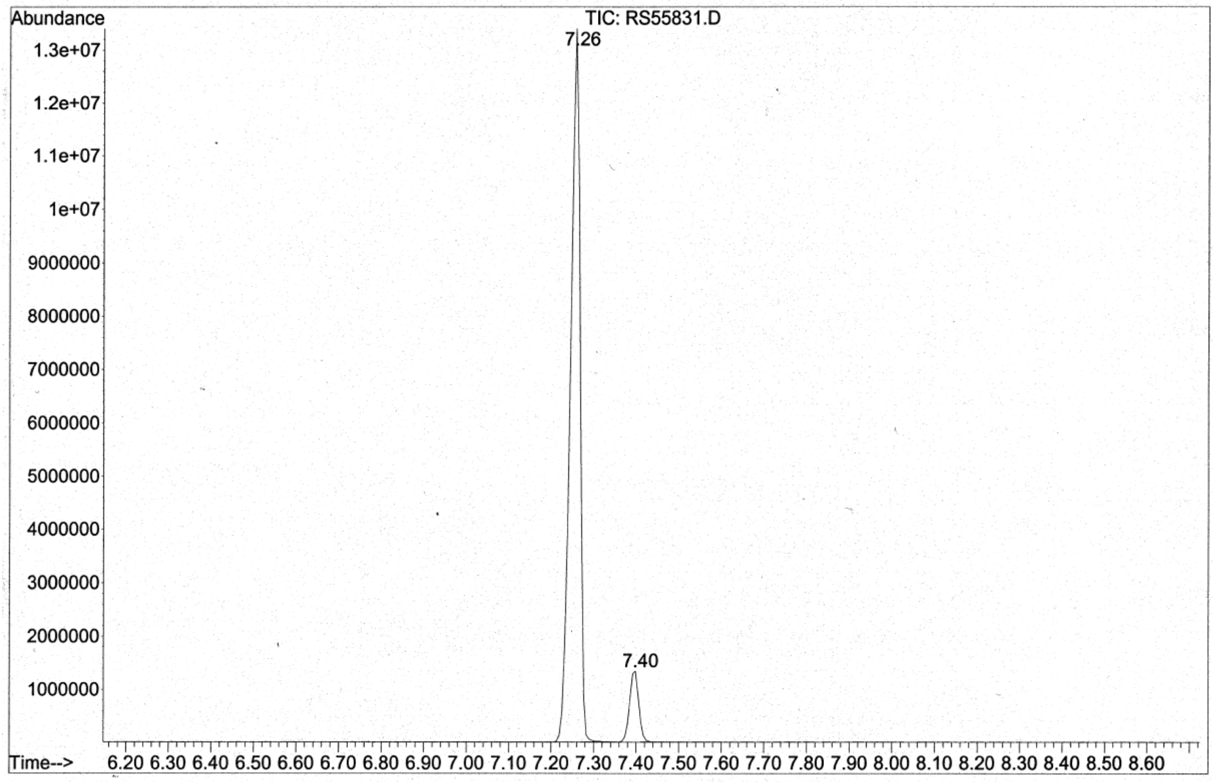

Figure S13. Chromatogram of the GC-MS analysis of purified 2-bromocyclohexanol as product of 2-bromocyclohexanone reduction with ThaADH. Enantiomers/diastereomers peaks at 7.26 min and 7.40 min, respectively.

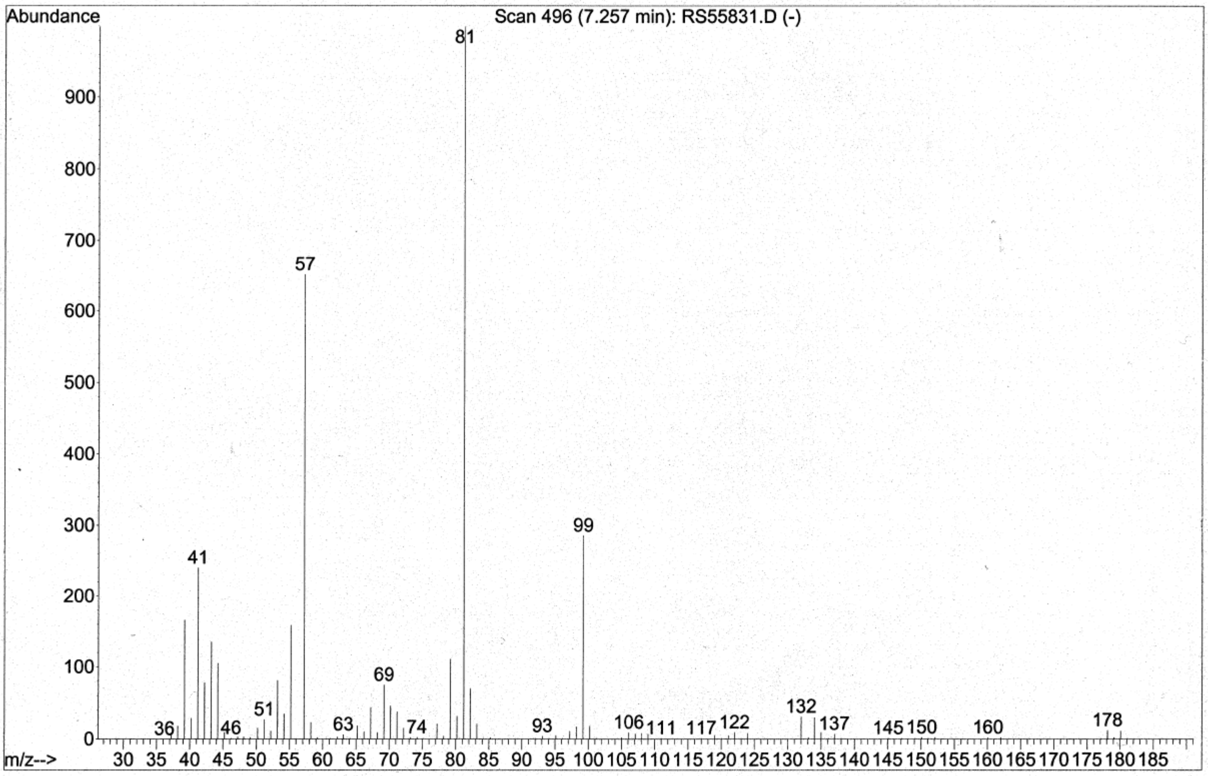

Figure S14. Mass spectrum of purified 2-bromocyclohexanol as product of the ThaADH catalyzed reduction of 2-bromocyclohexanone. Compound was eluted at 7.26 min (Figure S14). GC-MS (m/z): 180 (M+2), 178 (M^+).

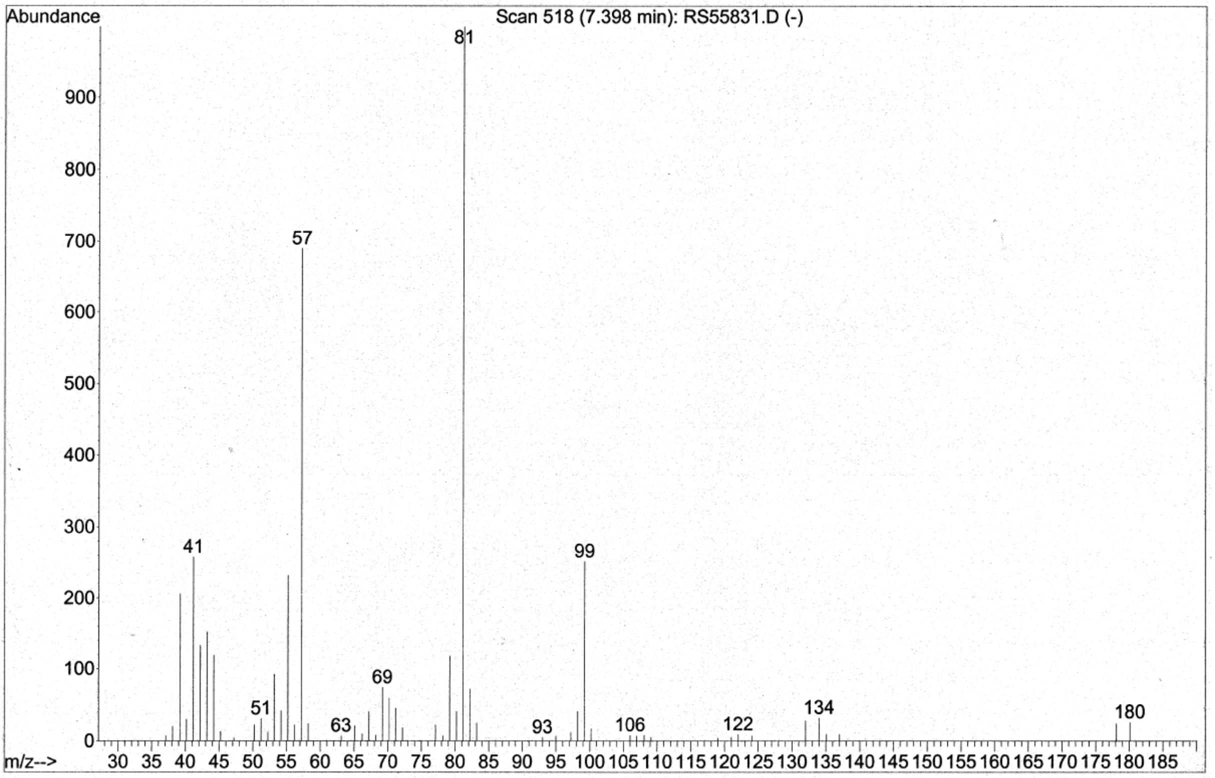

Figure S15. Mass spectrum of purified 2-bromocyclohexanol as product of 2-bromocyclohexanone reduction with ThaADH. Compound was eluted at 7.40 min (Figure S14). GC-MS (m/z): 180 (M+2), 178 (M^+).

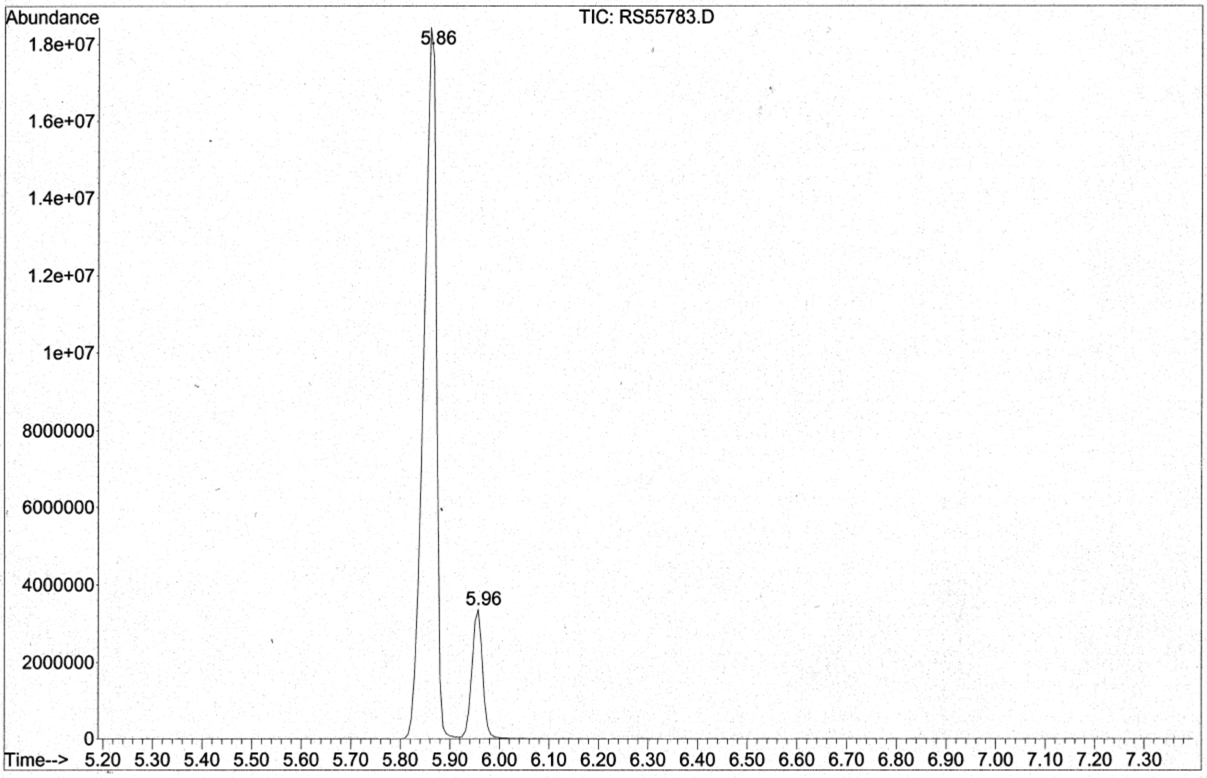

Figure S16. Chromatogram of the GC-MS analysis of purified 2-chlorocyclohexanol as product of 2-chlorocyclohexanone reduction with ThaADH. Enantiomers/diastereomers peaks at 5.86 min and 5.96 min, respectively.

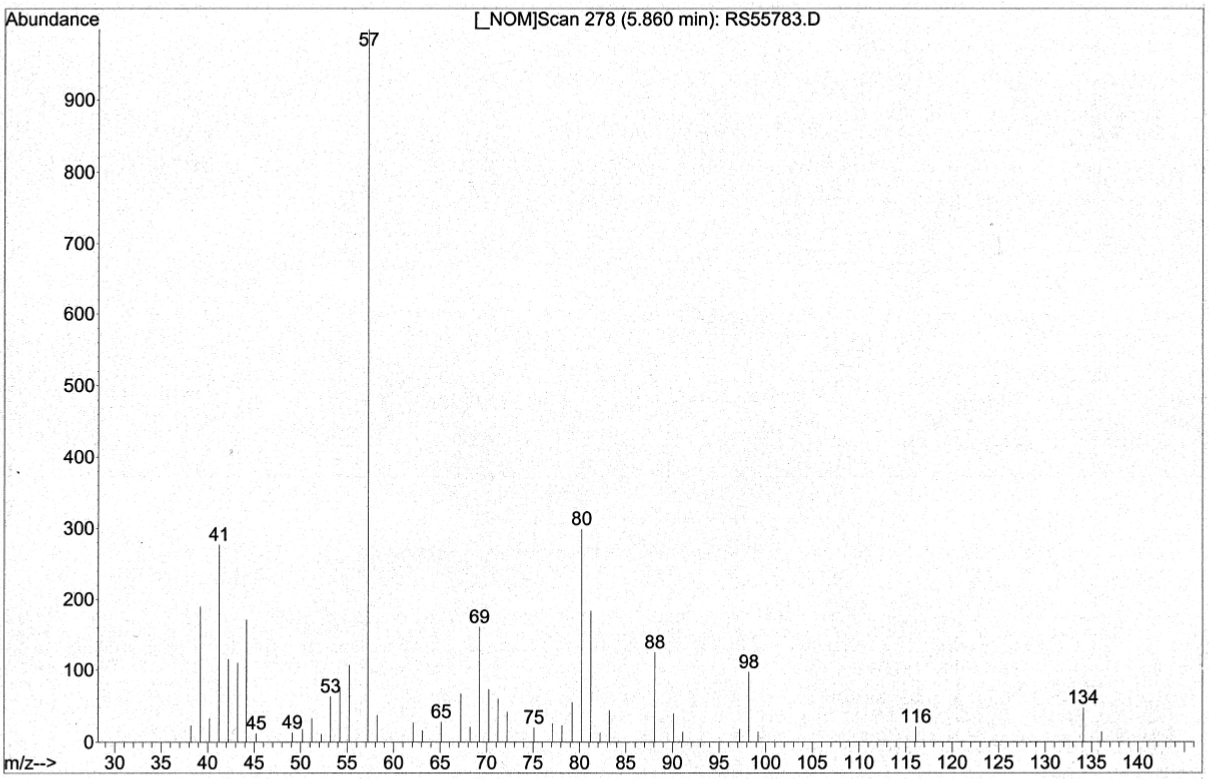

Figure S17. Mass spectrum of purified 2-chlorocyclohexanol as product of 2-chlorocyclohexanone reduction with ThaADH. Compound was eluted at 5.86 min (Figure S17). GC-MS (m/z): 136 (M+2).

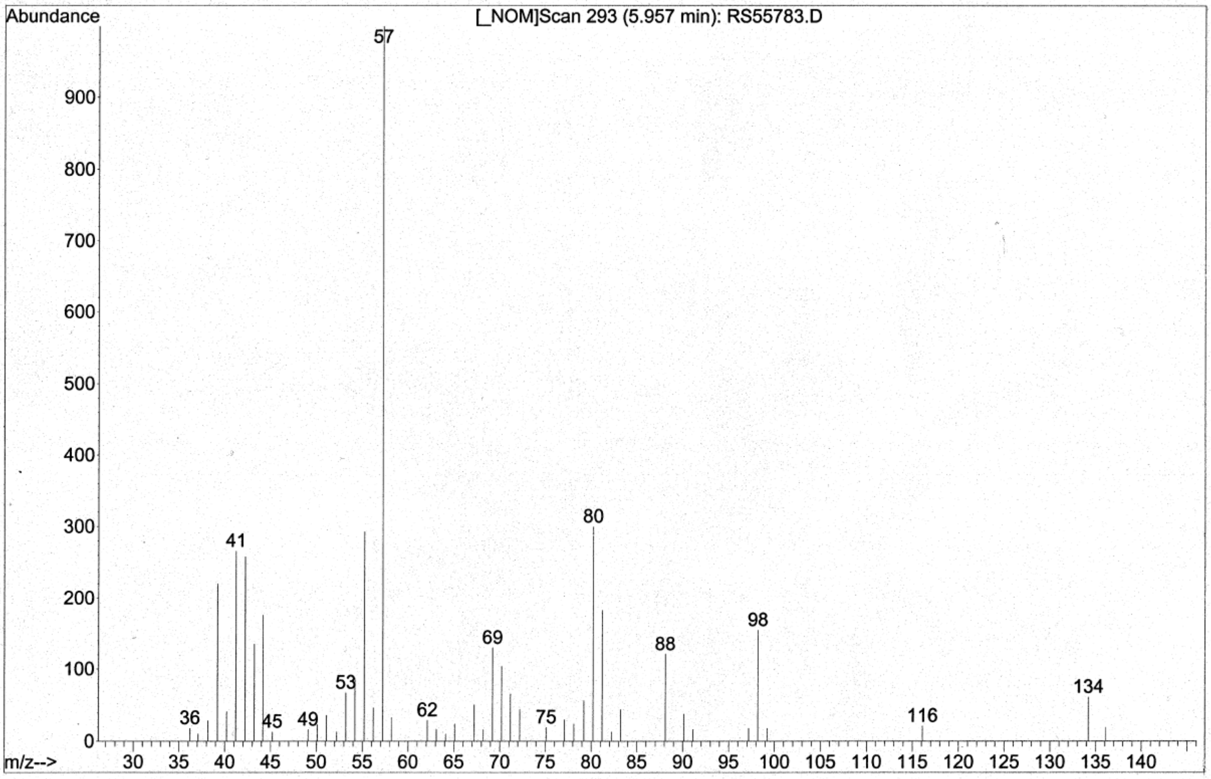

Figure S18. Mass spectrum of purified 2-chlorocyclohexanol as product of 2-chlorocyclohexanone reduction with ThaADH. Compound was eluted at 5.96 min (Figure S17). GC-MS (m/z): 136 (M+2).

Table S2. Atomic absorption analysis for enzymatic zinc detection.

c_{Zn} [mg L^{-1}]	c_{Enzyme} [mg mL^{-1}]	Zn per monomer
23.5	8.9	1.5
